# PIWIL2/PDK1 Axis Promotes the Progression of Cervical Epithelial Lesions via Metabolic Reprogramming to Maintain Tumor‐Initiating Cell Stemness

**DOI:** 10.1002/advs.202410756

**Published:** 2024-11-05

**Authors:** Yuebo Li, Wenhui Wang, Dongkui Xu, Haiyan Liang, Huan Yu, Ying Zhou, Jing Liang, Heming Sun, Xiaodie Liu, Ming Xue, Bin Ling, Dingqing Feng

**Affiliations:** ^1^ Department of Obstetrics and Gynecology China‐Japan Friendship Hospital Beijing 100029 China; ^2^ China‐Japan Friendship Hospital (Institute of Clinical Medical Sciences) Chinese Academy of Medical Sciences & Peking Union Medical College Beijing 100730 China; ^3^ VIP Department National Cancer Center/National Clinical Research Center for Cancer/Cancer Hospital Chinese Academy of Medical Sciences & Peking Union Medical College Beijing 100021 China; ^4^ Department of Obstetrics and Gynecology The First Affiliated Hospital of University of Science and Technology of China Hefei 230001 China; ^5^ Institute of Clinical Medical Sciences China‐Japan Friendship Hospital Beijing 100029 China

**Keywords:** cervical lesion, pyruvate dehydrogenase kinase 1, stemness, tumor‐initiating cells

## Abstract

When PIWIL2 expression is restored via heterogeneous integration of human papillomavirus, cellular reprogramming is initiated to form tumor‐initiating cells (TICs), which triggers cervical squamous intraepithelial lesions (SIL). TIC stemness is critical for the prognosis of SIL. However, the mechanisms underlying TIC stemness maintenance and tumorigenicity remain unclear. Here, it is revealed that aberrant pyruvate dehydrogenase kinase 1 (PDK1) expression is closely related to aerobic glycolysis in SIL and poor survival in patients with cervical cancer. Mechanistically, that PIWIL2, which induced by stable transfection of either PIWIL2 or HPV16 oncogene E6 in human primary cervical basal epithelial cells and keratinocyte cell line HaCaT, upregulates PDK1 expression via the LIN28/let‐7 axis, hence reprogramming metabolism to activate glycolysis and synchronize with TIC formation. It is further demonstrate that PDK1 is critical for TIC stemness maintenance and tumorigenicity via the PI3K/AKT/mTOR pathway both in vitro and in vivo, revealing a previously unclear mechanism for SIL progression, regression or relapse. Therefore, this findings suggest a potential rationale for prognostic predictions and selecting targeted therapy for cervical lesions.

## Graphical abstract

Heterogeneous integration of HPV restores PIWIL2 expression to cause TIC formation via initiating cellular reprogram. This study is the first to verify that PIWIL2 upregulates PDK1 expression, hence reprogramming metabolism to maintain TIC stemness and tumorigenicity via the PI3K/AKT/mTOR pathway both in vitro and in vivo, highlighting a potential prognostic prediction and therapeutic target for cervical lesions.

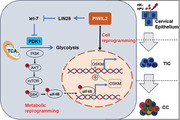

## 1 Introduction

Cervical cancer (CC) is one of the most common cancers in women worldwide; more than 600 000 new cases and 340 000 deaths caused by CC are reported each year.^[^ ^1^ ^]^ CC is closely related to infection with high‐risk human papillomavirus (HR‐HPV), as viral DNA integration into the host genome causes the development of cervical squamous intraepithelial lesions (SIL). SIL can be divided into low‐ and high grades of severity, i.e., LSIL and HSIL, respectively; the higher the lesion grade, the greater the risk of progression to invasive cancer.^[^ ^2^ ^]^ Therefore, cervical conization is the preferred treatment for these lesions but may seriously impair the fertility of a female patient. However, according to studies in clinical cohorts, some of these lesions can spontaneously regress, with a 61% regression probability for LSIL but only 28% for HSIL.^[^ ^3^ ^]^ Unfortunately, the mechanism underlying the choice of fate between progression or regression of cervical lesions remains unclear.

PIWIL2 is a member of the Piwi subfamily of the Argonaute family who play an important role in germline and stem cell maintenance, post transcriptional regulation, and a type of cancer‐testis antigen.^[^ ^4^ ^]^ As a stem cell protein, its aberrant expression has been verified in various precancerous lesions and cancers.^[^ ^5^ ^]^ Our previous studies revealed that E6 and E7 of HR‐HPV reactivated PIWIL2 expression, which sequentially initiated cellular reprogramming in the cervical basal epithelial cells (HCBC) via upregulating reprogramming factors (OSKM: OCT4, SOX2, KLF4, and C‐MYC), eventually resulting in tumor‐initiating cell (TIC) formation.^[^ ^6^ ^]^ TICs share several features with normal stem cells; for example, they can self‐renew and produce progeny cells with differentiation capacity.^[^ ^7^ ^]^ Moreover, TICs are predisposed to drive tumor progression, metastatic spread and therapeutic resistance throughout the tumor.^[^ ^7^, ^8^ ^]^ Therefore, TIC stemness is critical for the regression, persistence, progression, and relapse of SIL.

Accumulating evidence indicates that energy metabolism plays an important role in regulating stem cell functions and fates. Pluripotent stem cells have been verified to primarily utilize glycolysis for energy production, whereas normal cells rely on oxidative phosphorylation.^[^ ^7^, ^9^ ^]^ High glycolytic flux is common in various stem cell populations and is critical for pluripotency acquisition and maintenance. Similarly, TICs also exhibit a noticeable glycolytic phenotype characterized by upregulated glycolytic enzyme expression, elevated glucose uptake, high lactate production and ATP levels, and a significant decrease in mitochondrial oxidative metabolism.^[^ ^9^, ^10^ ^]^ In contrast, glycolysis inhibition causes TICs to be unable to maintain stemness, a weakness that may provide a potential therapeutic strategy and reduce the risk of metastasis and recurrence.^[^ ^11^ ^]^ Moreover, as normal somatic cells are reprogrammed into induced pluripotent stem cells, metabolic reprogramming from mitochondrial respiration to glycolysis occurs synchronously, indicating the close link between metabolic reprogramming and stemness.^[^ ^12^ ^]^

Pyruvate dehydrogenase kinase 1 (PDK1) is a gatekeeper enzyme involved in altered glucose metabolism in tumors. It promotes glycolytic metabolism via negatively regulating pyruvate dehydrogenase (PDH) and thereby inhibiting pyruvate entry into the tricarboxylic acid (TCA) cycle.^[^ ^11^, ^13^ ^]^ PDK1 has been verified to play a key role in enhancing cellular reprogramming and tumor progression.^[^ ^11^, ^14^ ^]^ Therefore, we can speculate that PDK1 participates in determining the outcome of SIL via controlling TIC maintenance. Hence, the underlying mechanism deserves further investigation.

In this study, we investigated the molecular mechanism via which PIWIL2‐initiated metabolic reprogramming synchronizes with TIC formation and glycolytic metabolism to maintain TIC stemness. Our data revealed that PIWIL2 upregulated PDK1 expression via the LIN28/let‐7 axis, causing a shift in cellular metabolism from oxidative phosphorylation to glycolysis and the sequential activation of PI3K/AKT/mTOR signaling, which promoted the expression of core transcription factors C‐MYC, NANOG, OCT4, SOX2, and KLF4, therefore maintaining TIC stemness via eIF4B phosphorylation. Thus, PDK1 may be a potential therapeutic target for treating SIL and CC.

## 2 Results

### 2.1 PDK1‐Mediated Glycolysis Correlates with the Progression of Cervical Lesions

**Figure 1:**
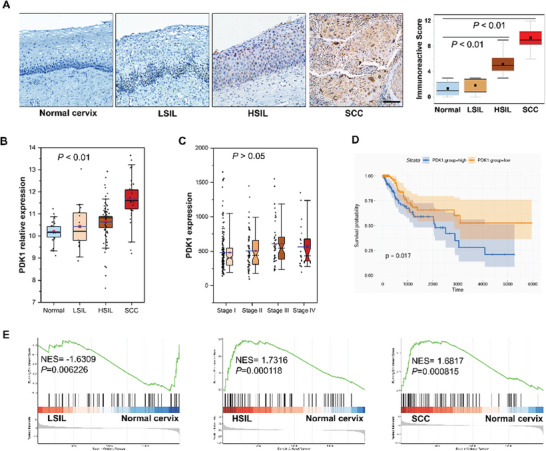
PDK1‐mediated glycolysis is correlated with cervical lesion progression. A) PDK1 expression was evaluated via immunohistochemical staining of cervical specimen sections from normal cervix, LSIL, HSIL, and SCC tissues; immunoreactivity scores are presented as the means ± SDs. B,E) Bioinformatic analysis of microarray data revealed the relationship of PDK1 gene expression with the degree of cervical lesion severity B) and with glycolysis gene set enrichment, determined using Gene Set Enrichment Analysis, in cervical lesions E). C,D) Analysis of The Cancer Genome Atlas cohort revealed the association of PDK1 expression with the cancer stage C) and survival of patients with CC D). PDK1, pyruvate dehydrogenase kinase 1; LSIL, low‐grade squamous intraepithelial lesions; HSIL, high‐grade squamous intraepithelial lesions; SCC, squamous cervical cancer.

PDK1 is a gatekeeper enzyme involved in altered glucose metabolism in tumors, and its expression is strongly correlated with tumor stage, degrees of invasion and metastasis, and overall survival.^[^ ^11b^ ^]^ Bioinformatic analysis of the microarray dataset GSE63514 revealed that the PDK1 gene expression level gradually increased with increasing lesion severity during the progression of cervical lesions (**Figure** **1**B). This finding was further verified by immunohistochemical staining of PDK1 in cervical specimen sections (Figure 1A), in which the PDK1 expression level was strongly correlated with lesion severity (R = 0.848, *P* < 0.01). Analyzing The Cancer Genome Atlas cohort revealed that patients with squamous CC (SCC) in the high PDK1 expression group had a significantly worse survival prognosis than those in the low PDK1 expression group (*P* = 0.017) (Figure 1D); however, PDK1 expression was not significantly correlated with the cancer stage in patients with SCC (*P* > 0.05) (Figure 1C). To further investigate the molecular functions of PDK1 in cervical lesions, Gene Set Enrichment Analysis was performed based on Gene Ontology and Kyoto Encyclopedia of Genes and Genomes gene sets. The REACTOME_GLYCOLYSIS gene set was enriched in the high PDK1 expression groups in HSIL in SCC tissues but not in LSIL (Figure 1E), indicating that high PDK1 expression in HSIL and SCC could activate glycolysis, hence reprogramming metabolism.^[^ ^15^ ^]^

### 2.2 PIWIL2 Initiates Cellular Metabolic Reprogramming via Upregulating PDK1 Expression

**Figure 2:**
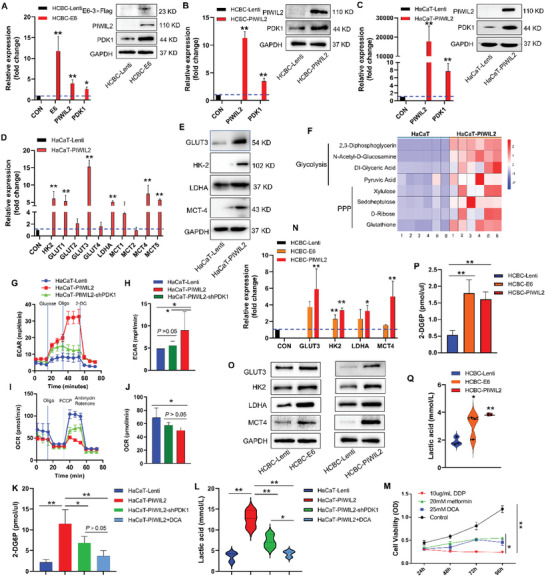
PIWIL2 initiates cellular metabolic reprogramming via upregulating PDK1 expression. A) Stable transfection with HPV16 E6 in HCBC significantly upregulated PIWIL2 and PDK1 in both mRNA and protein. B,C) The mRNA and protein expression of PDK1 increased significantly in HCBC B) and HaCaT C) cells transduced with the PIWIL2 lentiviral overexpression vector, respectively. D,E) The expression of glycolytic genes was measured using real‐time qPCR D) and western blotting E) in HaCaT cells stably expressing the empty lentiviral vector (HaCaT‐Lenti) or PIWIL2 (HaCaT‐PIWIL2). F) Metabolite abundances were determined using gas chromatography‐mass spectrometry to reveal metabolic state switch in HaCaT‐Lenti and HaCaT‐PIWIL2 cells. G–J) ECAR G,H) and OCR I,J) were measured with a Seahorse XF Analyzer in HaCaT‐Lenti, HaCaT‐PIWIL2, and HaCaT‐PIWIL2 cells transduced with the lentiviral vector expressing shPDK1 (HaCaT‐PIWIL2‐shPDK1). K,L) Glucose uptake K) and lactate production L) were measured in HaCaT‐Lenti, HaCaT‐PIWIL2, and HaCaT‐PIWIL2 cells in which PDK1 was silenced with shRNA (HaCaT‐PIWIL2‐shPDK1) or inhibited with 25 mM DCA (HaCaT‐PIWIL2+DCA). M) HaCaT‐PIWIL2 cell viability was analyzed using a CCK8 assay after treatment with 25 mM DCA, 20 mM metformin or 10 µg mL^−1^ DDP. N,O) The expression of glycolytic genes was measured using real‐time qPCR (N) and western blotting O) in HCBC stably transfected with E6 (HCBC‐E6), PIWIL2 (HCBC‐PIWIL2), or empty lentiviral vector (HCBC‐Lenti). The data are presented as the means ± SDs. *p < 0.05, **p < 0.01. ECAR, extracellular acidification rate; OCR, oxygen consumption rate; DCA, dichloroacetate; DDP, diamminedichloroplatinum; qPCR, quantitative polymerase chain reaction; HCBC, human cervical basal epithelial cells.

Our previous study confirmed that the reactivation of PIWIL2 expression by HR‐HPV oncoproteins initiated cellular reprogramming, subsequently leading to TIC formation and cervical tumorigenesis.^[^ ^6b^ ^]^ In this study, our data showed that PIWIL2 overexpression in HCBC cells and the normal keratinocyte cell line HaCaT via lentiviral transduction significantly upregulated PDK1 expression (**Figure** **2**B,C). HPV16 oncogene E6 stable transfection also induced PIWIL2 expression, which upregulated PDK1 in HCBC (Figure 2A); further, glycolytic genes (such as hexokinase 2 (HK2), glucose transporter (GLUT), lactate dehydrogenase A (LDHA), and monocarboxylate transporter (MCT)) were upregulated (Figure 2D,E), indicating that glycolytic metabolism was activated.

To investigate the change in metabolic state, we performed metabolomic analysis using gas chromatography‐mass spectrometry in HaCaT cells with or without PIWIL2 overexpression. The data showed that the abundances of metabolites of both the glycolysis pathway (pyruvic acid, DI‐glyceric acid, 2,3‐diphosphoglycerin, etc.) and pentose phosphate pathway (PPP; xylulose, sedoheptulose, D‐ribose, etc.) were markedly increased in HaCaT cells that stably overexpressed PIWIL2 (Figure 2F; Figure S1, Supporting Information). However, when PDK1 was knocked down in HaCaT‐PIWIL2 cells, the PIWIL2‐induced metabolic state switch was blocked (Figure S2, Supporting Information).

To verify the metabolomic analysis results, we performed glycolysis and mitochondrial stress tests using a Seahorse XF Analyzer in HaCaT‐PIWIL2 cells with or without sequential small hairpin RNA (shRNA)‐mediated PDK1 silencing. As expected, PIWIL2 overexpression significantly increased the extracellular acidification rate (ECAR), whereas PDK1 knockdown decreased the induced ECAR (Figure 2G,H). Conversely, compared with that in control HaCaT cells, the oxygen consumption rate (OCR) in cells with PIWIL2 overexpression markedly decreased; this decrease was reversed by PDK1 knockdown, but the OCR remained low (Figure 2I,J). Glucose uptake and lactate release were examined with enzymatic assay kits, and both increased significantly in PIWIL2‐overexpressing HaCaT cells, but decreased after PDK1 was knocked down by specific shRNA or blocked by PDK1‐specific inhibitor dichloroacetate (DCA; Figure 2K,L). Therefore, we evaluated the inhibition of proliferation via targeting cellular glycolysis in PIWIL2‐overexpressing cells. HaCaT‐ PIWIL2 cell proliferation was significantly inhibited by treatment with either DCA or metformin, which selectively inhibited the catalytic function of HK2 (Figure 2M). In HCBC, overexpression of either oncoprotein E6 or PIWIL2, same as that in HaCaT‐ PIWIL2 cells, also upregulated glycolytic genes (Figure 2N,O) and increased glucose uptake (Figure 2P) and lactate release (Figure 2Q).

### 2.3 PDK1 Maintains the Stemness of Reprogrammed Cells via a PIWIL2‐Initiated Process

**Figure 3:**
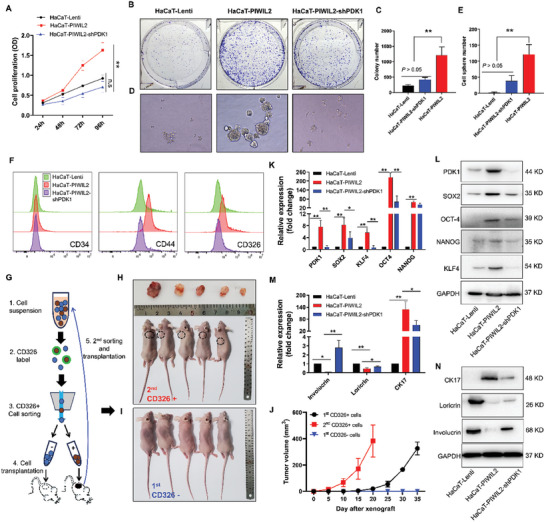
PDK1 maintains cell stemness with reprogramming initiated by PIWIL2. A) Proliferation of HaCaT‐Lenti, HaCaT‐PIWIL2 and HaCaT‐PIWIL2‐shPDK1 cells was measured using a CCK8 assay. B,C) Colony formation was observed B) and the number of colonies C) was compared among HaCaT‐Lenti, HaCaT‐PIWIL2, and HaCaT‐PIWIL2‐shPDK1 cells. D,E) Suspended cell spheres were observed D) and the number of cell spheres E) was determined in the HaCaT‐Lenti, HaCaT‐PIWIL2, and HaCaT‐PIWIL2‐shPDK1 cell groups. F) The expression of TIC marker CD326, CD44, and CD34 was analyzed using FACS in the HaCaT‐Lenti, HaCaT‐PIWIL2 and HaCaT‐PIWIL2‐shPDK1 cells. G–J) Tumorigenicity of CD326+ cells was evaluated via serial transplantation in nude mice. The flowchart shows the process involved in evaluating tumorigenicity of sorted CD326+ cells with serial transplantation G). CD326+ cells secondly sorted from xenografts formed apparent tumors H,J) whereas no tumors were observed in CD326‐ cells transplanted mice I). K,L) The expression levels of transcription factors associated with stem cells were measured via real‐time qPCR K), and protein levels were measured via western blotting L) in HaCaT‐Lenti, HaCaT‐PIWIL2, and HaCaT‐PIWIL2‐shPDK1 cells. M,N) The expression of the cervical epithelial stem cell marker CK‐17 and epithelial differentiation markers involucrin and loricrin were measured using real‐time qPCR M) and western blotting N) in HaCaT‐Lenti, HaCaT‐PIWIL2, and HaCaT‐PIWIL2‐shPDK1 cells. The data are presented as the means ± SDs. *p < 0.05, **p < 0.01. TIC, tumor‐initiating cells; PDK1, pyruvate dehydrogenase kinase 1; qPCR, quantitative polymerase chain reaction.

We further investigated the function of PDK1 in controlling the properties of cells with PIWIL2‐mediated reprogramming. Stable overexpression of PIWIL2 markedly promoted cell proliferation, whereas sequential PDK1 knockdown significantly inhibited growth (*P* < 0.01) (**Figure** **3**A). Both the adherent and suspended colony formation abilities of HaCaT‐PIWIL2 cells were significantly decreased by PDK1 knockdown (*P* <0.01) (Figure 3B–E). PIWIL2 stable transfection in HaCaT cells increased the expression of TIC marker CD44 and CD326 (EpCAM) and the ratio of side population (SP) cells, whereas the shRNA of PDK1 blocked this effect (Figure 2F; Figure S4A,B, Supporting Information). TIC tumorigenicity was evaluated via serial transplantation in immunodeficiency mice. A total of 5 × 10^6^ CD326+ cells were sorted using fluorescence‐activated cell sorting (FACS) and subcutaneously transplanted in nude mice. Tumors were formed in the CD326+ cells and cells secondly sorted from xenografts, whereas no tumor formation was observed in CD326‐ cells transplanted mice until 60 days after transplantation (Figure 3G–J). These CD326+ cells in formed tumors partially differentiated into CD326‐ (Figure S4C, Supporting Information) and CD31+ cells (Figure S4D, Supporting Information).

Consistent with our previous results demonstrating that PIWIL2 initiated cellular reprogramming via upregulating cellular reprogramming factors, such as OCT4, SOX2, KLF4, C‐MYC, and NANOG, to promote TIC formation, Gene Set Enrichment Analysis of the GSE63514 dataset revealed a greater enrichment of stem cell‐related gene sets, particularly WONG_EMBRYONIC_STEM_CELL_CORE, in specimens from patients with LSIL, HSIL, and SCC, than in normal cervix specimens, whereas the stem cell differentiation‐related gene set BOSCO_EPITHELIAL_DIFFERENTIATION_MODULE was significantly depleted in all the cervical lesion specimens (Figure S3, Supporting Information), indicating that the status of TICs was critical for the prognosis of cervical lesions. In the present study, upregulation of KLF4, NANOG, OCT4, and SOX2—similar to PDK1—was further verified in HaCaT‐ PIWIL2 cells at both the transcriptional (Figure 3K) and translational (Figure 3L) levels. PDK1 knockdown in HaCaT‐PIWIL2 cells clearly downregulated the expression of these cellular reprogramming factors (Figure 3K,L). Cytokeratin 17 (CK17), a TIC marker in the cervix, was induced by PIWIL2 overexpression but abolished by PDK1 silencing; expressions of the epithelial differentiation markers involucrin and loricrin were conversely altered by PDK1 modulation (Figure 3M,N), which may contribute to tumor suppression via targeting PDK1. Similarly, HCBC stably transfected with either E6 or PIWIL2 also exhibited apparent upregulation of these cellular reprogramming factors and stem cell marker CK17 (Figure S10A,B, Supporting Information).

To verify the tumor suppression of PDK1 on tumorigenicity in vivo, 5 × 10^6^ cells in the exponential growth phase were transplanted subcutaneously into the dorsal surface of nude mice. Consistent with our previous study,^[^ ^6b^ ^]^ HaCaT‐PIWIL2 cells formed evident tumors whereas HaCaT cells exhibited no tumorigenicity in these mice. However, unexpectedly, the mice transplanted with HaCaT‐PIWIL2 cells with the PDK1 knockdown also did not develop palpable tumors during the six‐week period (Figure S5A–C, Supporting Information). Compared with HaCaT cells, the xenografts derived from HaCaT‐PIWIL2 cell transplantation exhibited higher protein levels of PDK1, C‐MYC, SOX2, and phosphorylated STAT3 (Figure S5D, Supporting Information).

### 2.4 PDK1 Plays a Key Role in Determining the Glycolytic Phenotype of CC Cells

**Figure 4:**
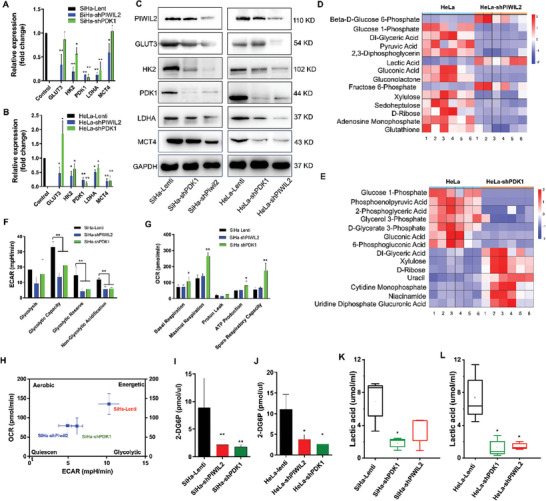
PDK1 plays a key role in determining the glycolytic phenotype of cervical cancer cells. A,B) Expressions of glycolytic genes GLUT3, HK2, PDK1, LDHA, and MCT4 were measured using real‐time qPCR in SiHa A) and HeLa B) cells stably transduced with the empty, shPIWIL2 or shPDK1 lentiviral vector. C) Protein levels of GLUT3, HK2, PDK1, LDHA, and MCT4 were measured using western blotting. D,E) Metabolites were analyzed with gas chromatography‐mass spectrometry to reveal the switch in metabolic state in HeLa cells with stable silencing of PIWIL2 D) or PDK1 E). F–H) ECAR F) and OCR G) were measured using a Seahorse XF Analyzer to determine the metabolic state H) of SiHa cells after stable transduction of the empty, shPIWIL2 or shPDK1 lentiviral vector. I–L) Glucose uptake in SiHa (I) and HeLa J) cells and lactate production in SiHa K) and HeLa J) cells were measured after stable transduction with the empty, shPIWIL2, or shPDK1 lentiviral vector. The data are presented as the means ± SDs. *p < 0.05, **p < 0.01. PDK1, pyruvate dehydrogenase kinase 1; ECAR, extracellular acidification rate; OCR, oxygen consumption rate; GLUT3, glucose transporter 3; HK2, hexokinase 2; LDHA, lactate dehydrogenase A, MCT4, monocarboxylate transporter 4.

Key glycolytic enzymes that could be novel targets for cancer therapy must be identified.^[^ ^16^ ^]^ In this study, PDK1 knockdown significantly downregulated glycolytic enzymes, including HK2, LDHA, and MCT4, but not GLUT3, in both HeLa and SiHa cells. The levels of these glycolytic enzymes decreased significantly after PIWIL2 knockdown in CC cells (**Figure** **4**A–C). To verify the role of PDK1 in controlling the glycolytic phenotype of CC cells, metabolomic analysis was performed using gas chromatography‐mass spectrometry. Metabolomic analysis results showed that the abundances of the glycolysis pathway metabolites significantly decreased but those of the PPP, some nucleotide‐related molecules (cytidine, uracil, etc.), and niacinamide evidently increased after PDK1 knockdown in HeLa cells (Figure 4E; Figure S6, Supporting Information). The abundances of the glycolysis pathway and PPP metabolites markedly decreased; however, glucose 6‐phosphate, fructose 6‐phosphate, and lactic acid accumulated in PIWIL2‐silenced HeLa cells (Figure 4D, Figure S6, Supporting Information).

The ECAR and OCR were measured by a Seahorse XF Analyzer to determine the metabolic state. The ECAR assay data showed that the glycolytic capacity, glycolytic reserve, and non‐glycolytic acidification significantly decreased after either PIWIL2 or PDK1 knockdown in SiHa cells (Figure 4F). The OCR assay data showed that both basal and maximal respiration, ATP production, and the spare respiratory capacity markedly increased in PDK1‐silenced SiHa cells, whereas no significant changes were observed in these parameters after PIWIL2 silencing (Figure 4G). These results further demonstrated that PDK1 was the key enzyme involved in maintaining glycolytic activity in CC (Figure 4H). Glucose uptake and lactic acid production decreased after PIWIL2 and PDK1 knockdown in SiHa and HeLa cells, respectively, which was consistent with the switch in metabolic state (Figure 4I–L).

### 2.5 Glycolytic Metabolism Regulates CC Cell Differentiation and Tumorigenicity

**Figure 5:**
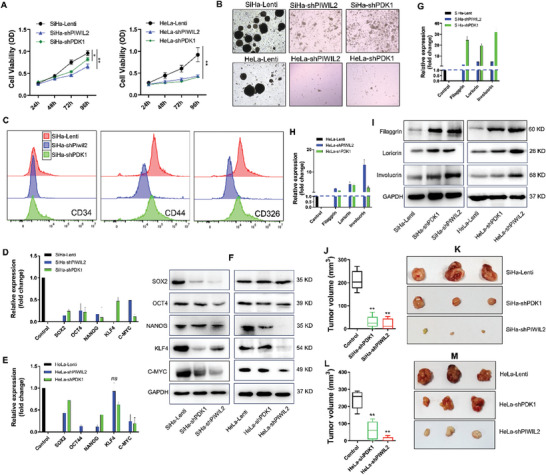
Glycolytic metabolism regulates the differentiation and tumorigenicity of cervical cancer cells. A) PIWIL2 or PDK1 was stably knocked down in SiHa and HeLa cells, after which cell growth was measured via a CCK8 assay. B) The ability of SiHa and HeLa cells to form suspended cell spheres was examined after stable knockdown of PIWIL2 or PDK1. C) The expression of TIC markers CD326, CD44, and CD34 was analyzed using FACS in SiHa‐Lenti, SiHa‐shPIWIL2, and SiHa‐shPDK1 cells. D–F) The transcript levels of SOX2, OCT4, NANOG, KLF4, and C‐MYC were measured via real‐time qPCR D,E) and protein levels were measured via western blotting F) in SiHa and HeLa cells after stable knockdown of PIWIL2 or PDK1. G–I) The transcript and protein levels of the epithelial differentiation markers filaggrin, loricrin and involucrin were measured using real‐time qPCR G,H) and western blotting I) in SiHa and HeLa cells after stable knockdown of PIWIL2 or PDK1. J–M) SiHa and HeLa cells with stably silenced PIWIL2 or PDK1 were transplanted subcutaneously into nude mice to evaluate tumorigenicity, after which tumor formation (K, M) and tumor volume J,L) were measured. The data are presented as the means ± SDs. *p < 0.05, **p < 0.01. PDK1, pyruvate dehydrogenase kinase 1; qPCR, quantitative polymerase chain reaction; TIC, tumor‐initiating cell.

As we previously reported, PIWIL2 knockdown suppressed cell proliferation and in vivo tumorigenicity in CC,^[^ ^6b^ ^]^ and PDK1 silencing similarly significantly inhibited cell growth and cell sphere formation (**Figure** **5**A,B). The proportion of CD44+ and CD326+ cells sharply decreased after PIWIL2 knockdown in SiHa, whereas only a slight decline was observed in PDK1‐silenced SiHa cells (Figure 5C). Expressions of transcription factors involved in cellular reprogramming, such as OCT4, SOX2, KLF4, C‐MYC, and NANOG, were significantly downregulated, which consequently caused the loss of cellular stemness (Figure 5D–F). A clear upregulation of cell differentiation markers, such as filaggrin, loricrin and involucrin, was observed after either PIWIL2 or PDK1 knockdown (Figure 5G–I). Moreover, the in vivo tumorigenicity of both HeLa and SiHa cells with either PIWIL2 or PDK1 silencing was clearly reduced (Figure 5J–M). Overall, these results demonstrate that PDK1 may be a target for CC therapy via blocking glycolysis.

### 2.6 Targeting PDK1 Enhances the Therapeutic Effect of DDP on CC

**Figure 6:**
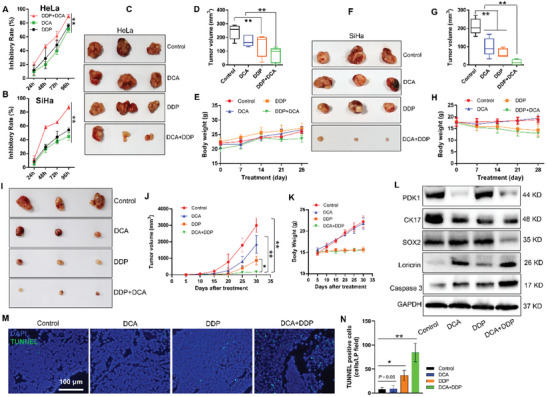
Targeting PDK1 enhances the therapeutic effect of DDP on cervical cancer. A,B) Growth inhibition of HeLa A) and SiHa B) cells treated with only DCA, only DDP, or in combination. C–H) HeLa and SiHa cells were transplanted subcutaneously into nude mice to establish a xenograft model, and mice were treated with DCA and DDP alone or in combination. Then, tumor formation C,F), tumor volume D,G), and body weight were examined to evaluate therapeutic efficacy. I–N) CC tissues were transplanted subcutaneously into nude mice to establish a PDX model, and the mice were treated with the therapeutic regimen described above. Tumor formation I), tumor volume J), and body weight K) were measured. At the end of the treatment period, tumor specimens were harvested to evaluate the expression of stemness and differentiation markers via western blotting L), and to evaluate cellular apoptosis via a TUNEL assay M,N). The data are presented as the means ± SDs. *p < 0.05, **p < 0.01. DDP, diamminedichloroplatinum; DCA, dichloroacetate; PDK1, pyruvate dehydrogenase kinase 1; PDX, patient‐derived xenograft.

To evaluate the growth inhibition effect of targeting PDK1, CC cells were treated with the specific PDK1 inhibitor DCA alone or in combination with diamminedichloroplatinum (DDP). The growth inhibition rates were 70.79% and 44.93% in HeLa and SiHa cells, respectively, 96 h after treatment with 25 mM DCA, approximately approaching the efficiency of treatment with 2 µg mL^−1^ DDP, whereas the growth inhibition rate was ≈90% after treatment with DCA and DDP in combination (**Figure** **6**A,B). Cells treated with DCA and DDP for 48 h had a higher apoptosis rate than those treated with either of the drugs alone (Figure S7, Supporting Information); this increase in apoptosis could, to some extent, contribute to growth inhibition.

The *in* *vivo* antitumor effects of these drugs were investigated in a CC xenograft mouse model in which the mice were treated with DCA alone or in combination with DDP. The data showed that treatment with either DCA or DDP alone noticeably inhibited tumor growth, but showed no significant differences within them at the end of the therapeutic schedule. However, the combination treatment significantly suppressed tumor growth in the mouse models established with both HeLa (Figure 6C,D) and SiHa (Figure 6F,G) cells. The treatment did not cause overt toxicity in mice, as no significant decreases in body weight was observed (Figure 6E,H).

A patient‐derived xenograft (PDX) model was used to verify these therapeutic effects. The results in this model revealed that DCA treatment alone obviously inhibited tumor growth and that the combination treatment had a more notable therapeutic effect (Figure 6I,J). However, to some extent, DDP treatment resulted in little weight gain but no significant weight loss in the mice (Figure 6K). The tumor specimens were harvested, and protein expression was examined via western blotting. PDK1 expression was strongly suppressed by DCA; therefore, expressions of the stemness markers CK17 and SOX2 were downregulated, whereas that of the cell differentiation marker loricrin was upregulated (Figure 6L). DCA treatment also induced cellular apoptosis, particularly in combination‐treated tumors, as the cleaved caspase 3 level (Figure 6L) and TUNNEL‐positive cell numbers (Figure 6M,N) evidently increased.

### 2.7 PIWIL2 Upregulates PDK1 Expression via the LIN28/let‐7 Axis

**Figure 7:**
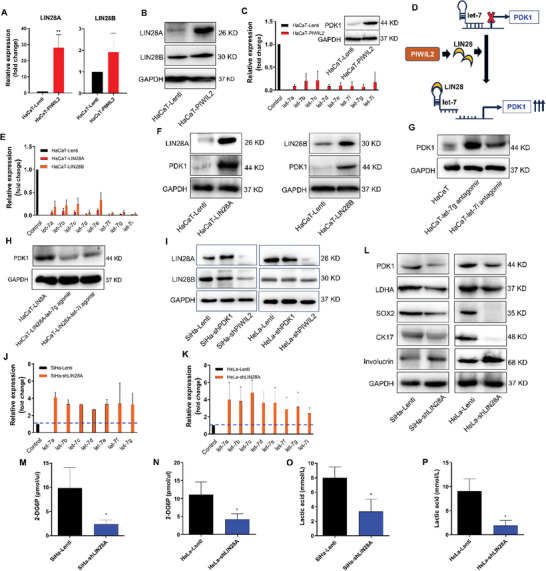
PIWIL2 upregulates PDK1 expression via the LIN28/let‐7 axis. A,B) LIN28A and LIN28B mRNA expression was measured using real‐time qPCR A) and protein levels were measured using western blotting B) in HaCaT and HaCaT‐ PIWIL2 cells. C) Real‐time qPCR analysis of let‐7 family member expression and western blot analysis of PDK1 expression were performed in HaCaT cells after stable PIWIL2 overexpression. D) Diagrammatic sketch of PIWIL2 upregulating PDK1 expression via the LIN28/let‐7 axis. E,F) Real‐time qPCR analysis of let‐7 family member expression E) and western blot analysis of PDK1 expression F) were performed in HaCaT cells stably transfected with LIN28A or LIN28B. G,H) PDK1 expression was measured via western blotting in HaCaT cells transfected with the let‐7 g or let‐7i antagomir G) and in HaCaT‐LIN28A cells transfected with the let‐7 g or let‐7i agomir H). I) PIWIL2 and PDK1 were knocked down using shRNA in SiHa and HeLa cells, after which the expression of LIN28A and LIN28B was measured via western blotting. J,K) Let‐7 family member expression was measured using real‐time qPCR after LIN28A was knocked down in SiHa J) and HeLa K) cells. L) The expression levels of the key glycolytic enzymes PDK1 and LDHA, stemness‐related genes SOX2 and CK17, and epithelial differentiation marker involucrin were measured using western blotting in SiHa and HeLa cells after stable transduction with the empty or shLIN28A lentiviral vector. M–P) Glucose uptake M,N) and lactate production O,P) were measured in SiHa and HeLa cells after LIN28A knockdown. The data are presented as the means ± SDs. *p < 0.05, **p < 0.01. PDK1, pyruvate dehydrogenase kinase 1; qPCR, quantitative polymerase chain reaction; LDHA, lactate dehydrogenase A.

The LIN28/let‐7 axis has been reported to facilitate aerobic glycolysis to promote cancer progression.^[^ ^14b^ ^]^ In this study, PIWIL2 overexpression in HaCaT cells induced LIN28 upregulation, particularly that of LIN28A (**Figure** **7**A,B), and PDK1 (Figures 2A and 7C), which also were observed in HCBC stably transfected with PIWIL2 or E6 (Figure 2A,B and Figure S10A,B, Supporting Information). The members of the let‐7 family (let‐7a, let‐7b, let‐7c, let‐7d, let‐7e, let‐7f, let‐7 g, and let‐7i) were markedly downregulated in HaCaT‐PIWIL2 compared with that in HaCaT‐Lenti (Figure 7C). Therefore, we supposed that PIWIL2 upregulated PDK1 via the LIN28/let‐7 axis to initiate metabolic reprogramming (Figure 7D). To further verify these results, stable transfection of either LIN28A or LIN28B into HaCaT cells was also shown to significantly decrease the expression of let‐7 family members (Figure 7E), increase PDK1 expression (Figure 7F), and subsequent upregulation of LDHA, CK17, and SOX2 (Figure S8A, Supporting Information). Treatment with let‐7 g and let‐7i antagomirs, which specifically blocked let‐7 g and let‐7i expression, also upregulated PDK1, LDHA, and CK17 expression in HaCaT cells (Figure 7G; Figure S8B, Supporting Information), whereas treatment with let‐7 g and let‐7i agomirs, which partially mimicked let‐7 g and let‐7i expression, downregulated PDK1 and LDHA but upregulated involucrin in LIN28A‐overexpressing HaCaT cells (Figure 7H; Figure S8C, Supporting Information).

In CC cells, PDK1 knockdown had no apparent effect on LIN28A or LIN28B expression, whereas PIWIL2 knockdown led to a significant decrease in LIN28A expression and a slight decrease in LIN28B expression (Figure 7I). After LIN28A was stably silenced in both HeLa and SiHa cells, the expression of all let‐7 family members was upregulated more than 2‐fold (Figure 7J and K). Therefore, after LIN28A was silenced in CC cells, PDK1 expression obviously decreased; LDHA, SOX2, and CK17 expression subsequently decreased; involucrin expression increased (Figure 7L). Consistent with these findings, glucose uptake (Figure 7M,N) and lactic acid production (Figure 7O,P) both significantly diminished in both HeLa and SiHa cells upon LIN28A knockdown. These findings were further verified in HaCaT‐PIWIL2 cells after LIN28A was silenced (Figure S8D,E, Supporting Information). Therefore, in cells with PIWIL2‐mediated cellular reprogramming, LIN28 was reactivated and PDK1 was upregulated via the LIN28/let‐7 axis to reprogram metabolism and maintain stemness.

### 2.8 PDK1 Activates the PI3K/Akt/mTOR Pathway to Maintain Stemness

**Figure 8:**
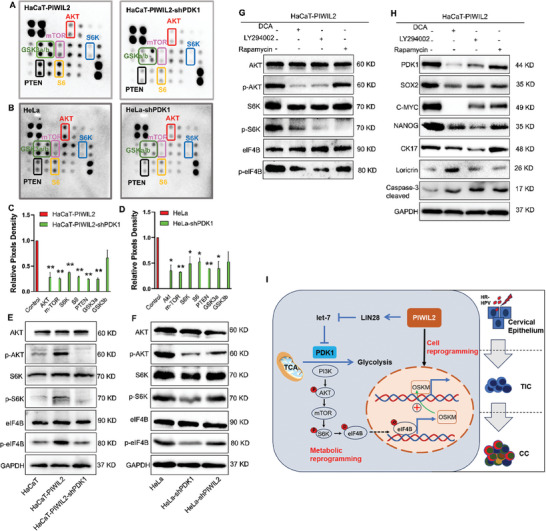
PDK1 activates the PI3K/AKT/mTOR pathway to maintain stemness. A–D) The functional status of PI3K‐AKT‐mTOR signaling was evaluated using a human phosphorylation pathway profiling array in HaCaT‐PIWIL2 A) and HeLa B) cells stably transduced with the empty or shPDK1 lentiviral vector. The phosphorylation levels of AKT, mTOR, S6K, S6, PTEN, and GSK3a/b were measured with normalized densitometry C,D). E,F) Phosphorylation of AKT, S6K and eIF4B was evaluated via western blotting in HaCaT‐PIWIL2 cells after PDK1 silencing E) and in HeLa cells after PIWIL2 or PDK1 silencing F). G,H) HaCaT‐PIWIL2 cells were treated with the PDK1 inhibitor DCA, PI3K/AKT inhibitor LY294002, or mTOR inhibitor rapamycin. Phosphorylation levels of PI3K‐AKT‐mTOR pathway proteins were evaluated via western blotting G). Western blotting was performed to measure levels of the key glycolytic enzyme PDK1; stemness‐related markers SOX2, C‐MYC, NANOG and CK17; epithelial differentiation marker loricrin; apoptosis‐related protein cleaved caspase 3 H). I) Summary of the results of this study: 4299 PIWIL2 upregulates PDK1 expression via the LIN28/let‐7 axis, which synchronizes with TIC formation, via initiating cellular reprogramming to reprogram metabolism and maintain the stemness of TICs through the PI3K‐AKT‐mTOR pathway, thus promoting cervical lesion progression. PDK1, pyruvate dehydrogenase kinase 1; TIC, tumor‐initiating cell; DCA, dichloroacetate.

As insulin‐PI3K‐mTOR signaling is highly evolutionarily conserved in the regulation of growth and glucose metabolism,^[^ ^17^ ^]^ we investigated the functional status of these target proteins using a human phosphorylation pathway profiling array (Figure S9, Supporting Information). The array data showed that Ser473 phosphorylation (p‐Ser473) of AKT, p‐Ser2448 of mTOR, p‐Thr421/p‐Ser424 of S6K, and p‐Ser235/236 of S6 ribosomal protein were markedly decreased in both HaCaT‐PIWIL2 and HeLa cells after shRNA‐mediated PDK1 knockdown (**Figure** **8**A,B), which could be quantitively exhibited with normalized densitometry (Figure 8C,D). These may indicate that the LIN28/let‐7/PDK1 axis promotes activation of the PI3K‐AKT‐mTOR pathway. Western blot analysis further verified that in HaCaT cells, PIWIL2 overexpression activated the PI3K‐AKT‐mTOR pathway, which was blocked by the subsequent PDK1 knockdown (Figure 8E) or treatment with specific PDK1 inhibitor DCA, PI3K/AKT inhibitor LY294002, or mTOR inhibitor rapamycin (Figure 8G). Similarly, the phosphorylation level of AKT and S6K also evidently increased in HCBC after being stably transfected with PIWIL2 (Figure S10C, Supporting Information). The inhibition of this pathway was also observed in HeLa cells when either PIWIL2 or PDK1 was silenced (Figure 8F). The expression of the stem cell factors SOX2, C‐MYC, NANOG, and CK17 decreased because of DCA, LY294002, or rapamycin treatment, whereas the protein levels of the differentiation marker loricrin and apoptosis marker cleaved caspase 3 increased (Figure 8H).

Overall, we speculate that PIWIL2 initiates cellular reprogramming that results in TIC formation while restoring PDK1 expression via the LIN28/let‐7 axis to synchronously activate a metabolic switch, which plays a critical role in maintaining TIC stemness via the PI3K‐AKT‐mTOR pathway (Figure 8H).

## 3 Discussion

The oncoprotein PIWIL2 is expressed during the embryonic stage, and its expression is lost after birth, except for in germ cells in the testis; however, its expression is restored in many types of cancer and precancerous lesions, predominantly in cancer stem cells (CSCs) and TICs.^[^ ^5^, ^6^, ^18^ ^]^ Our previous work demonstrated that PIWIL2, reactivated by E6 and E7 of HPV16, initiated cellular reprogramming that caused TIC formation via upregulating OCT4, SOX2, KLF4, C‐MYC, and NANOG, which led to cervical neoplastic lesions.^[^ ^6b^ ^]^ These results were further verified in this study: HPV16 oncogene E6 stably transfected HCBC, the target cell of HPV; it restored PIWIL2 expression and sequentially initiated cellular reprogramming, which could be the initial molecular event for cervical lesion (Figure 2; Figure S10, Supporting Information). Further, these cells were positive for CD44 and CD326 (Figure 3F), well‐known cancer stem cell markers,^[^ ^19^ ^]^ and exhibited an increasing proportion of SP (Figure S4A, Supporting Information) and tumorigenesis with sorted CD326+ cells for serial transplantation (Figure 3G–I), which could differentiate into both CD326‐ cancer cells and CD31+ vascular endothelial cells (Figure S4C,D, Supporting Information). Therefore, we believe that TIC formed from cellular reprogramming by PIWIL2 is the origin of cervical lesions.

A stemness component has been verified to be required for the development of neoplastic properties. Similar to embryonic stem cells (ESCs) and adult stem cells, TICs primarily depend on robust glycolytic activity to supply ATP to maintain stemness, therefore promoting highly tumorigenic behaviors.^[^ ^7^, ^9^, ^10^, ^20^ ^]^ In this study, after stable transfection of PIWIL2, both HCBC and HaCaT cells, bearing the characteristics of TICs,^[^ ^6^, ^19^ ^]^ exhibited: significantly increased expression of glycolytic enzymes, including PDK1, HK2, and LDHA; increased glucose uptake and lactic acid production, accompanied by upregulated expression of GLUT3 and MCT4, respectively; a marked metabolic switch from oxidative metabolism to glycolysis, as verified by Seahorse and metabolomics analyses (Figure 2). These findings indicated that PIWIL2 not only initiated cellular reprogramming to promote TIC formation but also synchronously triggered metabolic reprogramming to fulfill cellular bioenergetic and biosynthetic requirements, an event also considered necessary for successful cellular reprogramming.^[^ ^12^, ^14^ ^]^ In contrast, PIWIL2 knockdown in CC cells significantly downregulated stem cell pluripotency factors (OSKM: OCT4, SOX2, KLF4, C‐MYC) (Figure 5D–F) and subsequently decreased the expression levels of GLUT3, HK2, PDK1, LDHA and MCT4; therefore, glycolytic activity sharply decreased, whereas the abundances of TCA cycle metabolites increased (Figure 4; Figure S6, Supporting Information).

The expression of PDK1, which promotes glycolytic metabolism via inhibiting pyruvate entry into the TCA cycle, is consistently elevated in stem cells, such as ESCs, adult stem cells, induced pluripotent stem cells (iPSCs) and CSCs.^[^ ^11^, ^21^ ^]^ PDK1 plays critical roles in maintaining stemness and regulating tumor progression.^[^ ^11^, ^14^ ^]^ Therefore, an aberrant expression of PDK1 is frequently observed in tumors and, to some extent, contributes to tumor invasion, metastasis, drug resistance and poor patient survival.^[^ ^14^, ^22^ ^]^ In this study, PDK1 expression was noticeably elevated in HSIL and SCC, consistent with HPV DNA integration and PIWIL2 reactivation, and increased PDK1 expression was related to poor survival outcomes (Figure 1). PDK1 knockdown in HaCaT‐PIWIL2 cells significantly downregulated glycolytic enzymes, which decreased glycolytic activity, glucose uptake and the abundances of glycolytic metabolites. These alterations were accompanied by simultaneous decreases in stemness factor expression levels, which reduced colony and sphere formation in vitro and tumorigenicity in vivo (Figure S5, Supporting Information); however, cell differentiation was observed to be enhanced (Figure 3). Furthermore, PDK1 knockdown in CC cells significantly decreased glycolytic activity, sphere formation and tumorigenicity, whereas the expression levels of cell differentiation markers increased (Figures 4 and 5). These results demonstrate that PDK1 plays a critical role in TIC formation during the progression of cervical lesions via enhancing cellular reprogramming and maintaining stemness via initiating metabolic reprogramming. Cervical lesions containing TICs may have the potential for bidirectional differentiation into both benign and malignant lesions.^[^ ^5^, ^6^ ^]^ Therefore, PDK1 undoubtedly determines the choice of fate between regression or progression of cervical lesions.

Consistent with the upregulation of PDK1 expression, LIN28A and LIN28B expression increased and those of let‐7 family microRNAs decreased in HaCaT cells with PIWIL2‐mediated reprogramming (Figure 7A–C). When either LIN28A or LIN28B was overexpressed in HaCaT cells, the expression of the mature let‐7 family members let‐7a, ‐7b, ‐7c, ‐7d, ‐7e, ‐7f, ‐7 g, and ‐7i was significantly suppressed (Figure 7E), while the expression of PDK1 was increased (Figure 7F). In this regard, previous studies have also demonstrated that reactivation of LIN28 causes the downregulation of let‐7 microRNA family members and hence upregulates the downstream target PDK1 via the LIN28/let‐7 axis to regulate aerobic glycolysis.^[^ ^14^, ^17^, ^23^ ^]^ Forced expression of let‐7 g and ‐7i mimics in LIN28A‐overexpressing HaCaT cells markedly decreased PDK1 expression but increased the expression of the cell differentiation marker involucrin (Figure 7H; Figure S8C, Supporting Information); however, inhibition of let‐7 g and ‐7i by antagomir treatment increased the expression of PDK1 and the stem cell marker CK17, respectively (Figure 7G; Figure S8B, Supporting Information). Accordingly, in CC cells, knocking down LIN28 expression with shRNA, in contrast, upregulated let‐7 family members and downregulated glycolytic enzymes and stem cell pluripotency factors, hence significantly decreased glucose uptake and lactic acid production (Figure 7J–P). Overall, these findings indicate that LIN28/let‐7/PDK1 axis‐mediated metabolic reprogramming plays vital roles in maintaining the stemness of TICs initiated via PIWIL2.

LIN28A/B and let‐7 regulate PI3K‐AKT‐mTOR signaling to switch on metabolic reprogramming in cancer cells.^[^ ^14^, ^17^, ^24^ ^]^ We also verified PI3K‐AKT‐mTOR pathway activation in HaCaT cells and HeLa cells with PIWIL2‐mediated reprogramming. Moreover, Ser473 phosphorylation (p‐Ser473) of AKT and p‐Ser2448 of mTOR were markedly increased, and the mTOR target S6K and its downstream target eIF4B were also obviously phosphorylated (Figure 8A,B). Therefore, PI3K‐AKT‐mTOR signaling was notably inhibited via either PIWIL2 or PDK1 knockdown (Figure 8E,F). The phosphorylation status of eIF4B is important for its tumorigenic activity via promoting the transcription of C‐MYC, ODC, Cdc25C, etc.^[^ ^25^ ^]^ Blocking PDK1 and Akt activity with DCA and LY294002, respectively, decreased the phosphorylation levels of AKT, S6K and eIF4B accordingly. However, AKT phosphorylation was not inhibited by the mTOR inhibitor rapamycin. Blocking PDK1, AKT, and mTOR apparently downregulated stem cell factors, whereas involucrin and caspase 3 expression were elevated (Figure 8H). DCA inhibited the growth of CC cells both in vitro and in vivo and showed good therapeutic efficacy in the PDX model when administered either alone or in combination with DDP (Figure 6). Therefore, these results indicate that PDK1, regulated by the LIN28/let‐7 axis, promotes the transcription of stem cell factors via the PI3K‐AKT‐mTOR pathway, thus enhancing the tumorigenicity of TICs (Figure 8I).

Therefore, our study revealed that the oncofetal protein PIWIL2, whose expression was restored via HR‐HPV integration, initiated TIC formation and synchronously reactivated PDK1 expression via the LIN28/let‐7 axis to reprogram metabolism and maintain stemness via the PI3K‐AKT‐mTOR pathway. Thus, it promoted cervical lesion progression. Conversely, switching off metabolic reprogramming mediated by PDK1 abolished the tumorigenicity of TICs, hence contributing to cervical lesion regression. Consequently, PDK1 provides a novel therapeutic option for targeting TICs.

## 4 Experimental Section

### Clinical Samples

Forty‐one histological specimens, including tissues obtained during hysterectomy and loop/cone biopsy, were retrieved from the archives of the Department of Pathology of the China‐Japan Friendship Hospital. The specimens comprised of 10 normal cervix, 10 LSIL, 10 HSIL, and 11 CC tissues samples. Fresh normal cervical tissues from patients who underwent a hysterectomy with no cervical lesion or HPV infection were used for isolating HCBC and performing primary culture. A fresh tumor specimen from a patient with CC, aged 48 years, was obtained within 1 h after the hysterectomy to establish a PDX mouse model. The project was approved by the Ethics Committee of the China‐Japan Friendship Hospital (approval number: 2020‐28‐K20).

### Primary HCBC Isolation and Culture

A small piece of fresh cervix (≈1 mm^3^) was rinsed with DPBS (without Ca^2+^ or Mg^2+^) (14 190, Gibco, MA, USA) containing 1000 U mL^−1^ of penicillin and streptomycin, respectively, then digested with 250 U mL^−1^ dispase (17 105, Gibco) with 100 U mL^−1^ of penicillin/streptomycin for 18 h at 4 °C. After dispase digestion, the cervical epidermal layer was separated and further digested with 0.05% trypsin for a single‐cell suspension. Trypsin activity was stopped using a 10 mg mL^−1^ Soybean trypsin inhibitor (17 075, Gibco), after which the cell suspension was centrifuged and rinsed with DPBS twice. The HCBCs were gently resuspended with a complete serum‐free keratinocyte medium (10 744 019, Gibco) and cultured at 37 °C with 5% CO_2_. Upon reaching 60%–75% confluency, the cells were dislodged with trypsin at 37 °C for 5–10 min, further treated with the 10 mg mL^−1^ Soybean trypsin inhibitor to stop the trypsin reaction, and then passaged for culture.

### Cell Culture and Transfection

HeLa, SiHa, and HaCaT cells were purchased from the American Type Culture Collection (ATCC) and cultured in high‐glucose Dulbecco's modified Eagle's medium (DMEM; 41 965 062, Gibco, USA) supplemented with 10% fetal bovine serum (FBS; 16 000 044, Gibco), 100 U mL^−1^ penicillin and 100 mg mL^−1^ streptomycin at 37 °C with 5% CO_2_.

The lentiviral vectors pLenti‐CMV‐PIWIL2‐3Flag‐SV40‐EGFP‐neo, pLenti‐Ubi‐PDK1‐3Flag‐SV40‐puro, pLenti‐CMV‐HPV16E6‐3Flag‐EF1‐GFP‐t2a‐puro, pLenti‐CMV‐LIN28A‐3flag‐EF1‐mcherry‐t2a‐neo, and pLenti‐CMV‐LIN28B‐3flag‐EF1‐mcherry‐t2a‐neo were used to infect cells for overexpression of PIWIL2, PDK1, HPV16E6, LIN28A, and LIN28B, respectively. Cells transfected with the corresponding empty lentiviral vectors were used as the controls. For the knockdown of PIWIL2, PDK1, LIN28A, and LIN28B, cell lines were transfected with the shRNA lentiviral vectors pLenti‐shPIWIL2‐Ub‐EGFP‐IRES‐puro, pLenti‐U6‐shPDK1‐CMV‐puro, pLenti‐U6‐shLIN28A‐EF1‐mecherry‐t2a‐neo, and pLenti‐U6‐shLIN28B‐EF1‐mecherry‐t2a‐neo, respectively. Three specific shRNAs were designed for each target gene of PDK1, PIWIL2 and LIN28A, and the one with the highest knockdown efficiency was selected for further experiments (Table S1 and Figure S11, Supporting Information). The negative control was a lentiviral vector containing a scrambled shRNA sequence without specific gene degradation effects. Stably transduced cells were selected via culturing with G418 (A1720, SigmaAldrich, MO, USA) or puromycin (540 411, SigmaAldrich, USA) for two weeks.

### Reagents and Treatment

Cisplatin (DDP; 232 120), sodium dichloroacetate (DCA; 347 795) and metformin hydrochloride (317 240) were purchased from Sigma‒Aldrich. Cultured CC cells were treated with 2 µg mL^−1^ DDP and 25 mM DCA alone or in combination. HaCaT cells overexpressing PIWIL2 were treated with DDP or metformin at a concentration of 10 µg mL^−1^ or 20 mM, respectively. Cells were treated with 40 µM LY294002 (S1105, Selleck) to inhibit PI3K signaling or 100 nM rapamycin (S1039, Selleck) to inhibit mTOR signaling. The corresponding control cells were treated with vehicle alone. The hsa‐let‐7i‐3p mimic (miR10004585‐1‐5), hsa‐let‐7g‐5p mimic (miR10000414‐1‐5), hsa‐let‐7i‐3p inhibitor (miR20004585‐1‐5) and hsa‐let‐7g‐5p inhibitor (miR20000414‐1‐5) were purchased from RiboBio (Guangzhou, China) and transfected into cells using Lipofectamine 3000 (L3000015, Invitrogen, USA).

### Cell Proliferation Assay

Cells were seeded in 96‐well plates at a density of 1 × 10^3^ cells well^−1^. Cell viability was evaluated at 24, 48, 72, and 96 h using a WST‐8 Cell Counting Kit (CCK‐8; Dojindo, Japan) according to the manufacturer's instructions. All the experiments were performed independently in triplicate.

### Colony Formation

Cells were seeded into 6‐well plates at a density of 2000 cells well^−1^. For the colony formation assay in adherent culture, complete medium supplemented with 10% FBS was used, and the colonies were stained with 0.05% crystal violet after two weeks. For the suspended sphere formation assay, cells were cultured in serum‐free medium supplemented with 10 ng mL^−1^ bFGF (100‐18B, PeproTech), 20 ng mL^−1^ EGF (AF‐100‐15, PeproTech) and 5 µg mL^−1^ insulin (PHR8925, Sigma‒Aldrich). The medium was refreshed by adding one‐half of the initial volume every three days, and spheres were counted under a phase contrast microscope at the end of the three‐week culture period.

### RNA Isolation and Quantitative PCR

Total RNA was extracted using TRIzol (15 596 018, Invitrogen) and reverse transcribed to cDNA using Superscript III reverse transcriptase (2680, TaKaRa, Japan). Quantitative real‐time PCR (qRT–PCR) was performed using the GoTaq qPCR Master Mix Kit (A6002, Promega, USA) on an ABI7500 real‐time PCR instrument (Thermo Fisher). The primer sets used were listed in Table S2 (Supporting Information). For qRT‒PCR analysis of let‐7 miRNA family members, we used the Mir‒X miRNA qRT‒PCR TB Green Kit (638 314, Clontech) as described by the manufacturer. The specific primer sequences used for let‐7 first‐strand synthesis and qRT‒PCR analysis are listed in Table S3 (Supporting Information).

### Antibodies and Western Blot Analysis

Cell and tumor tissue samples were lysed in RIPA buffer (P0013C, Beyotime, China) supplemented with protease inhibitor cocktail (P1005, Beyotime) and phosphatase inhibitor cocktail (P1045, Beyotime). Lysates were loaded and proteins separated on a 10% polyacrylamide gel (D0174S, Beyotime) in 5 × SDS–PAGE sample loading buffer, and the separated proteins were transferred to a polyvinylidene fluoride (PVDF) membrane (IPFL00010, Merck Millipore, Germany). The membrane was blocked for 1 h in TBST containing 5% milk and subsequently incubated with the indicated primary antibodies overnight at 4 °C. After incubation with species‐specific HRP‐conjugated secondary antibodies (Cell Signaling) at room temperature for 1 h, protein bands were detected with a chemiluminescent substrate (34 080, Thermo). The primary antibodies used for immunoblotting were listed in Table S4 (Supporting Information).

### Immunohistochemistry

Cervical tissues from patients and tumor tissues from the mouse PDX model were fixed with 4% paraformaldehyde and subsequently embedded in paraffin. Then, the slides were dewaxed with xylene and rehydrated through a series of washes with decreasing concentrations of ethanol, and antigen retrieval was performed with 0.01 M citrate buffer (pH 6.0) in a microwave oven for 10 min. After cooling to room temperature, the slides were incubated with 3% H_2_O_2_ for 10 min to inactivate endogenous peroxidase. The slides were incubated overnight at 4 °C in a humidified chamber with specific primary antibodies (Table S4, Supporting Information). The binding of the primary antibodies was visualized using a ChemMate detection kit (PV‐9000, ZSBIO, China). The slides were lightly counterstained with Mayer's hematoxylin for 30 s and were then dehydrated in increasing concentrations of ethanol before two sequential 10 min incubations in xylene prior to mounting.

Immunoreactivity was evaluated semiquantitatively. The immunoreactivity score was calculated as the intensity score × proportion score. The intensity score was defined as follows: 0, negative; 1, weak; 2, moderate; or 3, strong staining. The proportion score was defined as follows: 0, 0%; 1, <10%; 2, 11–50%; 3, 51–80%; or 4, >80% positive cells. The total score ranged from 0 to 12. Two different pathologists evaluated all the specimens in a blinded manner.

### TUNNEL Assay

Tumor tissues from the mouse PDX model were fixed with 4% paraformaldehyde and subsequently embedded in paraffin. After deparaffinization, the sections were washed and permeabilized for 5 min on ice with 0.1% Triton X‐100 and were then incubated with 20 µg mL^−1^ proteinase K (ST532, Beyotime) for 15 min at room temperature. TUNNEL was performed using an in situ apoptosis detection kit (C1088, Beyotime) according to the manufacturer's directions. The sections were covered with TUNNEL reaction mix (equilibration buffer, FITC‐dUTP, TdT enzyme) and incubated at 37 °C for 1 h in a humidified chamber. The sections were further labeled with streptavidin‐FITC in the dark for 30 min. After counterstaining with DAPI (2 µg mL^−1^), TUNNEL‐positive cells were counted from fluorescence images acquired in 10 random fields at 200 × magnification.

### SP Cell Analysis

A single‒cell suspension (10^6^ cells mL^−1^) was incubated in 1 × PBS containing 5 µg mL^−1^ Hoechst 33 342 (14 533, Sigma–Aldrich) and 2% FBS at 37 °C for 1 h with gentle agitation every 15 min. Control cells were incubated with an additional 50 mmol L^−1^ verapamil (1 711 202, Sigma–Aldrich). After incubation, cells were washed with ice‐cold 1 × PBS containing 2% FBS. SP analysis was performed using flow cytometry (FACSCalibur, BD Bioscience, NJ, USA) with 355 nm exciting light and 450/20 BP filter for blue fluorescence and 675 EFLP for red fluorescence.

### FACS Analysis

An Annexin V‐633/PI Apoptosis Detection Kit (AD11, Dojindo, Japan) was used for detection of cellular apoptosis following the manufacturer's instructions. In brief, CC cells were harvested after treatment with 2 µg mL^−1^ DDP and 25 mM DCA alone or in combination for 48 h. The cells were resuspended in 1 × Annexin V binding buffer and were then labeled with Annexin V‐633 and propidium iodide (PI) for 15 min in the dark at room temperature. Subsequently, the cells were washed, resuspended in 1 × PBS supplemented with 2% FBS, and analyzed via flow cytometry (FACSCalibur, BD).

For analyzing of the expression of stem cell markers, cells were stained with the following antibody cocktail: anti‐CD326‐PE, anti‐CD44‐PE‐cy7, anti‐CD34‐APC, and anti‐MHCI‐PE‐cy7 (369 805, 103 029, 343 607, 311 430, BioLegend, CA, USA). Cells were then washed, resuspended in 1 × PBS with 2% FBS, and analyzed using flow cytometry (FACSCalibur).

### Glucose Uptake Assay

Cells were seeded in 96‐well plates at a concentration of 1 × 10^4^ cells well^−1^ and cultured in serum‐free medium. After 48 h, the medium was replenished with glucose‐free medium containing 100 µM 2‐deoxy‐D‐glucose (2‐DG) and incubated for 2 h at 37 °C. 2‐DG is metabolized to 2‐DG‐6‐phosphate (2‐DG6P), which was further oxidized to generate NADPH, and the level of NADPH was determined with a glucose uptake assay kit (ab136955, Abcam) according to the manufacturer's instructions.

### Lactate Production Assay

Cells were seeded and cultured in 96‐well plates at a density of 1 × 10^4^ cells well^−1^ in complete medium supplemented with 10% FBS for 48 h at 37 °C. The extracellular lactate concentration was measured using 200 µL of the supernatant with a Lactate Colorimetric Assay Kit (D799851‐0050; Sangon Biotech, China) according to the manufacturer's instructions.

### Extracellular Metabolic Flux Analysis

The oxygen consumption rate (OCR) and extracellular acidification rate (ECAR) were measured using an XF96 Analyzer system (Seahorse Bioscience) following the manufacturer's instructions. In brief, cells were plated at a density of 2000 cellswell^−1^ in an XF96 Cell Culture Microplate (Seahorse, #102601‐100). The following day, the adherent cells were washed, and the medium was changed to XF Base Medium (Seahorse, #103575‐100) supplemented with 1 mM L‐glutamine (Seahorse, #103579‐100) and 1 mM pyruvate (Seahorse, #103578‐100) (mitochondrial stress test) or with 1 mM L‐glutamine and 10 mM glucose (Seahorse, #103577‐100) (glycolysis stress test) and was equilibrated for 1 h at 37 °C without a CO_2_ supply. For OCR measurements, oligomycin, FCCP, rotenone and antimycin (1 µM) were injected sequentially at specific time points. For ECAR measurement, 10 mM glucose, 2 µM oligomycin and 100 mM 2‐DG were added sequentially at specific time points. After all the measurements were completed, the cells were dissociated and counted.

### Untargeted Metabolomic Analysis

Six replicates of each group of cells (4 × 10^6^ cells) were prepared and subjected to gas chromatography–mass spectrometry (GC–MS) for detection of glycolytic metabolites, TCA cycle metabolites, nucleotide pools, amino acids, peptides, and lipids, as guided by OE Biotech Co., Ltd. (Shanghai, China). Samples were thawed at room temperature for 1 h, after which 1 mL of an ice‐cold mixture of methanol and H_2_O (4:1, vol/vol) was added. After the mixture was vortexed for 1 min, metabolites were extracted by ultrasonication for 20 min in an ice‐water bath and then stored overnight at −40 °C. The extract was centrifuged at 4 °C (12 000 rpm) for 10 min. A total of 400 µL of the supernatant in a glass vial was concentrated in a centrifugal freeze dryer. Next, 80 µL of 15 mg mL^−1^ methoxylamine dihydrochloride in pyridine was added, and the mixture was vortexed for 1 h at 37 °C. Then, 50 µL of N,O‐bis(trimethylsilyl)trifluoroacetamide (BSTFA; containing 1% trimethylchlorosilane (TMCS)), 20 µL of n‐hexane and 10 µL of 10 alkanes (C8/C9/C10/C12/C14/C16/C18/C20/C22/C24, dissolved in chloroform) were added, and the mixture was vortexed vigorously for 2 min and then derivatized for 1 h at 70 °C. Additionally, samples for quality control (QC) were prepared by mixing small equal aliquots of each sample and then extracting metabolites as described above.

Untargeted metabolomic analysis was performed on the Agilent 7890B‐5977A platform. In brief, a 1 µL aliquot of each derivative was injected onto a DB‐5MS capillary column (Agilent J&W Scientific, Folsom, CA, USA) in splitless mode. The injection temperature was maintained at 260 °C. The column temperature was kept at 60 °C for 30 s; was then increased sequentially to 125 °C at 8 °C min^−1^, 210 °C at 8 °C min^−1^, 270 °C at 15 °C min^−1^, and 305 °C at 20 °C min^−1^; and held at 305 °C for 5 min. Helium was used as the carrier gas, with a flow rate of 1 mL min^−1^. The quadrupole temperature was set at 150 °C, and the ion source temperature was set at 230 °C. The mass scanning range was m/z 50–500 at a rate of 30 spectra/s after a solvent delay of 5 min.

### Human AKT Pathway Phosphorylation Array Analysis

A human AKT phosphorylation antibody array (AAH‐AKT‐1, RayBiotech, USA) was used according to the manufacturer's instructions to simultaneously determine the relative levels of 18 phosphorylated AKT pathway proteins in cell lysates. In brief, the membranes were blocked in blocking buffer for 30 min at room temperature and were then incubated with the samples overnight at 4 °C. The samples were then decanted, and the membranes were washed with washing buffer and incubated in detection antibody cocktail for 2 h overnight at 4 °C. After 5 washes, the membranes were incubated with HRP‐conjugated anti‐rabbit IgG at room temperature for 2 h, sequentially washed thoroughly with washing buffer, and exposed to chemiluminescence detection buffer in the dark. High‐resolution images were acquired with a ChemiDoc XRS Imaging System (Bio‐Rad, USA), after which the signal intensities were further analyzed.

### Bioinformatics Analysis

The original microarray data of cervical lesions (GSE63514), comprising data for 24 normal cervix specimens, 14 CIN1 lesion specimens, 22 CIN2 lesion specimens, 40 CIN3 lesions, and 28 cancer specimens, were downloaded from the Gene Expression Omnibus (GEO) database (https://www.ncbi.nlm.nih.gov/geo/). The platforms used to generate these data sets are based on GPL570 and the Affymetrix Human Genome U133 Plus 2.0 Array. The limma package was used to standardize the gene chip data and analyze differences in expression. To investigate the role of PDK1‐triggered glycolysis in cervical lesions, gene set enrichment analysis (GSEA) was performed with the package ClusterProfiler based on the REACTOME_GLYCOLYSIS gene set. Differences with a *P* value of < 0.05 and an adjusted *P* (p.adjust) value of < 0.05 were considered significant.

For survival analysis, the CC dataset was obtained from The Cancer Genome Atlas (TCGA) (https://portal.gdc.cancer.gov/). This dataset contains data related to 252 cervical squamous cell carcinomas (SCCs) and 52 endocervical adenocarcinomas, along with the clinical data of the corresponding patients. The samples were divided into the low and high PDK1 expression groups by using R software. The hazard ratio (HR) was calculated via a Cox regression model, and survival curves were plotted from Kaplan‒Meier estimates. A *P* value of < 0.05 was considered to indicate statistical significance.

### Animal Studies

In vivo experiments were performed in accordance with the institutional guidelines for the use of laboratory animals (approval number: zryhyy61‐23‐03‐03). Four‐week‐old female BALB/c nude mice (Beijing Charles River Laboratory Animal Technology Co., Ltd., Beijing, China) were fed in a pathogen‐free animal facility for at least one week before use. To assess the sequential tumorigenicity of TICs, 5 × 10^6^ CD326+ cells were sorted by FACS from HaCaT‐PIWIL2 cells and transplanted subcutaneously into the dorsal surface of each mouse. Then, as the tumors formed, mice were sacrificed, and xenograft tumors were resected. The xenograft tumors were digested into single‐cell suspension and CD326+ cells were resorted again using FACS for injection into another mouse. Meanwhile, 5 × 10^6^ CD326‐ cells were also subcutaneously injected into mice to evaluate tumorigenesis.

To observe the tumorigenicity of cells with PDK1 knockdown, 5 × 10^6^ cells in the exponential growth phase were harvested and injected subcutaneously into the dorsal surface of each mouse, after which tumor growth was continuously monitored. To evaluate the anti‐tumor efficacy of the PDK1 inhibitor DCA, mice were administered DCA (200 mg kg day^−1^) alone or in combination with DDP (2 mg kg^−1^, twice a week) after palpable tumors were established. Furthermore, once the CC PDX model was established as we previously described,^[^ ^26^ ^]^ the mice were treated with the same therapeutic schedule comprising DCA and DDP. Tumors were measured with calipers twice weekly, and the tumor volume was calculated as V = (length × width^2^)/2. At the end of the experiment, the mice were sacrificed, and the tumors were harvested and weighed.

### Statistical Analyses

The means and standard deviations of the mean (SDs) were calculated, and statistically significant differences were determined using one‐way analysis of variance or unpaired Student's *t* test (SPSS software, version 19.0, SPSS, Inc., USA). A *P* value of < 0.05 was considered statistically significant.

## Conflict of Interest

The authors declare no conflict of interest.

## Author Contributions

D.Q.F. and Y.B.L. designed experiments and wrote the manuscript; Y.B.L., W.H.W., Y.H., H.M.S. and X.M. performed experiments; D.K.X. and X.D.L. performed bioinformatics analysis; Y.Z. and H.Y.L. performed data analysis; D.Q.F., B.L. and J.L. conceived the study, supervised the experiments and wrote the manuscript.

## Supporting information



Supporting Information

## Data Availability

The data that support the findings of this study are available from the corresponding author upon reasonable request.
