# Supplementary material for: PIWIL2/PDK1 Axis Promotes the Progression of Cervical Epithelial Lesions via Metabolic Reprogramming to Maintain Tumor‐Initiating Cell Stemness
Source: Adv Sci (Weinh). 2024 Nov 5;11(48):2410756. doi: 10.1002/advs.202410756 (PMC11672288; doi:10.1002/advs.202410756)
Supplement: Supplementary file 1 — Supporting Information [file ADVS-11-2410756-s001.pdf]

ADVANCED SCIENCE
Open Access

Supporting Information

for *Adv. Sci.*, DOI 10.1002/advs.202410756

PIWIL2/PDK1 Axis Promotes the Progression of Cervical Epithelial Lesions via Metabolic Reprogramming to Maintain Tumor-Initiating Cell Stemness

Yuebo Li, Wenhui Wang, Dongkui Xu, Haiyan Liang, Huan Yu, Ying Zhou, Jing Liang, Heming Sun, Xiaodie Liu, Ming Xue, Bin Ling* and Dingqing Feng**

Table S1. The target sequences for shRNA lentiviral vector

Gene	Accession	No.	Target Seq
PIWIL2	NM_001135721	shRNA-1	CAACTATGAGATTCCTCAA
		shRNA-2	CAATGGAAAGAATTAACTT
		shRNA-3	ATATAACAATCGTACCTAT
PDK1	NM_001278549	shRNA-1	GCTCTGTCAACAGACTCAATA
		shRNA-2	CCAGGGTGTGATTGAATACAA
		shRNA-3	CCAAACTGCAATGTACTTGAA
LIN28A	NM_024674	shRNA-1	GGCAGTGGAGTTCACCTTTAA
		shRNA-2	CCTGGTGGAGTATTCTGTATT
		shRNA-3	TCGAGAGGAAGAAGAAGAAAT
LIN28B	NM_001004317	shRNA-1	TTGGATTCATCTCCATGATAA
		shRNA-2	TGTACACCAAAGCAAACTATT
		shRNA-3	TCCAAAGGCCTTGAGTCAATA

Table S2. Specific primer sequences for real-time RT-PCR

Gene name	Forward primer sequences (5'-3')	Reverse primer sequences (5'-3')	Product (bp)
PDK1	GCTGTATGGCCTGCAAGATGA	AACATTCTGGCTGGTGACAGGA	143
PDK2	AACCTGCTTCCCGACCGAGTGCTGA	AGTGAACTGGCTCAGGGTGCGATGG	135
PDK3	TGAACCCAGGGATGCTTCAA	AACAGTGAGTGGGACACCTTT	106
PDK4	CCTGTGAGACTCGCCAACAT	TCCACCAAATCCATCAGGCT	119
HK1	ATCAGGACACACTACGACAGACTGG	TTAAGATGTTGCGGACGATTTCAC	115
HK2	AATGGAGCGAGGTCTGAGCAA	CTCCAAGGTCCAAGGCCAAG	122
GLUT1	CTGCAACGGCTTAGACTTCGAC	TCTCTGGGTAACAGGGATCAAACA	101
GLUT2	GCTGCGAATAAACAGGCAGGA	CAGCACAGCAGTGATGACAGTGA	107
GLUT3	GCCTTTGGCACTCTCAACCAG	TCAGACCCAAGGATGAATTCCAG	86
GLUT4	GGGCTGAGACAGGGACCATAAC	CATGAGCAATGGCATCCAGAA	95
LDHA	CATGGCCTGTGCCATCAGTATC	TGCCAGAGACAATCTTTGGTGTTC	146
MCT1	TGTGGAATGCTGTCCTGTCCTC	TGATGCCCATGCCAATGAA	143
MCT2	CCTGCGCCAGAGACCAGATAA	TTGGCACCAAGAGTCCCAGAG	109
MCT4	AGGAGTTTGGGATCGGCTACAG	AAAGCGGTTCACGCACACAC	104
MCT5	TTATTTCTGGATGGGTTGCTGA	GGAAATGTGGTGGCTAAAGGAG	115
Filaggrin	CAATCTGAGGGCACTGAAAGG	CTAACACTTCCGTGCTGAGAG	125
Involucrin	TCCAGTCAATACCCATCAGG	TGCTCACATTCTTGCTCAGG	156
Loricrin	AGTGGACTGCGTGAAGAC	GCCAGAACCGCTGCTACC	112
KRT17	TGCAGAGTGGCAAGAGTGAG	TTCATGCTGAGCTGGGACTG	85
C-MYC	GCAGCTGCTTAGACGCTGGA	CGCAGTAGAAATACGGCTGCAC	133
KLF4	AAGAGTTCCCATCTCAAGGCACA	GGGCGAATTTCCATCCACAG	91
NANOG	CCTGTGATTTGTGGGCCTGA	CTCTGCAGAAGTGGGTTGTTTG	168
OCT4	GTGCCGTGAAGCTGGAGAA	TGGTCGTTTGGCTGAATACCTT	192
SOX2	GTGAGCGCCCTGCAGTACAA	GCGAGTAGGACATGCTGTAGGTG	82
GAPDH	CTTAGCACCCCTGGCCAAG	GATGTTCTGGAGAGCCCCG	151

Table S3. Specific primer sequences for let 7 first-strand synthesis and real-time RT-PCR

Name	Sequence
U6 RT	CTCAACTGGTGTCGTGGAGTCGGCAATTCAGTTGAGAAAAATATG
U6-F	CTCGCTTCGGCAGCACA
U6-R	AACGCTTCACGAATTTGCGT
let 7a/e/f RT	CTCAACTGGTGTCGTGGAGTCGGCAATTCAGTTGAGAACTATAC
let 7b RT	CTCAACTGGAGCTAGTTTCGTCGTAGGGCAGTTGAGAACCACAC
let 7c RT	GTCGTATCCAGTGCAGGGTCCGAGGTATTCGCACTGGATACGACAACCAT
let 7d RT	CTCAACTGGTGTCGTGGAGTCGGCAATTCAGTTGAGAACTATGC
let 7g RT	CTCAACTGGTGTCGTGGAGTCGGCAATTCAGTTGAGAACTGTAC
let 7i RT	GTCGTATCCAGTGCAGGGTCCGAGGTATTCGCACTGGATACGACAACAGC
let 7a/b/c-F	CCAGCTGGGTGAGGTAGTAGGTTGT
let 7d-F	CCAGCTGGGAGAGGTAGTAGGTTGC
let 7e-F	CCAGCTGGGTGAGGTAGGAGGTTGT
let 7f-F	CCAGCTGGGTGAGGTAGTAGATTGT
let 7g/i-F	CCAGCTGGGTGAGGTAGTAGTTTGT
let 7c/i-R	TCCAGTGCAGGGTCCGAGGTA
let 7a/d/e/f/g-R	CTGGTGTCGTGGAGTCGGCAATT
let 7b-R	CTGGAGCTAGTTTCGTCGTAGGG

Table S4 Antibodies applied in this study

Vendor	Antibodies	Clone number	Catalog number	Application	Working dilution
Abcam	PDK1	4A11	ab110025	IHC	1:500
				WB	1:1000
	GLUT1	EPR3915	ab115730	WB	1:5000
	HK2		ab227198	WB	1:2000
	LDHA		ab47010	WB	1:500
	SOX2	EPR3131	ab92494	IHC	1:100
				WB	1:1000
	OCT4	EPR17929	ab181557	IHC	1:500
				WB	1:1000
	NANOG		ab21624	WB	1:200
	KLF4	EPR3550(2)(ABC)	ab151733	WB	1:2000
	C-MYC	Y69	ab32072	WB	1:500
	LIN28A	EPR4640	ab124765	WB	1:5000
	LIN28B		ab71415	WB	1:500
	CK17		ab53707	IHC	1:100
				WB	1:500
	Loricrin		ab137533	WB	1:500
	Involucrin		ab53112	WB	1:500
	eIF4B	EP2299Y	ab68474	WB	1:10000
	p-eIF4B (S406)	EPR7683	ab134138	WB	1:10000
	GAPDH	EPR16891	ab181602	WB	1:5000
Cell Signaling Technology	GLUT3	E7M7V	40538	WB	1:1000

	AKT	C67E7	4691	WB	1:1000
	pAKT (Ser473)	193H12	4058	WB	1:1000
	STAT3	124H6	9139	WB	1:1000
	pSTAT3 (Tyr705)	D3A7	9145	WB	1:2000
	ERK1/2	137F5	4695	WB	1:1000
	pERK1/2 (Thr202/Tyr204)	D13.14.4E	4370	WB	1:2000
	P70 S6K		9202	WB	1:1000
	pP70 S6K (Thr421/Ser424)		9204	WB	1:1000
ThermoFisher	Filaggrin		PA5-116911	WB	1:1000
Santa Cruz	MCT4	D-1	sc-376140	WB	1:200
Bioss Antibodies	Caspase 3		bs-0081R	WB	1:500
Beyotime	CD31		AG2849	IF	1:100
BioLegend	CD326	CO17-1A	369805	Flow Cyt	
	CD44	IM7	103029	Flow Cyt	
	CD34	561	343607	Flow Cyt	
	HLA-A,B,C	W6/32	311430	Flow Cyt	

Supplementary Figure Legends

Figure S1. Metabolites abundances were measured using GC-MS in HaCaT cells with stable transfection of PIWIL2.

HaCaT-Lenti and HaCaT-PIWIL2 cells were subjected to GC−MS for metabolite detection. Untargeted metabolomic analysis was performed with base peak chromatogram (A), PCA (B), and PLS-DA (C). Some glycolytic metabolites were significantly elevated in HaCaT-PIWIL2 cells compared with HaCaT-Lenti cells (D). GC–MS, gas chromatography-mass spectrometry; PCA, principal component analysis; PLS-DA, Partial Least Squares Discrimination Analysis.

Figure S2. PDK1 regulates the types of metabolites produced in HaCaT cells overexpressing PIWIL2, using determined by GC–MS.

(A) Base peak chromatogram of HaCaT-PIWIL2 cells with or without PDK1 knockdown via shRNA. (B) PCA was used to determine the distribution of all samples and the stability of the analysis process. (C) OPLS-DA was used to distinguish the differences in the metabolites. (D) Heatmap showing the levels of significantly differentially abundant metabolites. (E-F) Boxplot showing the different yields of metabolites in the glycolysis (E) and pentose phosphate (F) pathways. PDK1, pyruvate dehydrogenase kinase 1; GC−MS, gas chromatography-mass spectrometry; PCA, Principal component analysis; PLS-DA, Partial Least Squares Discrimination Analysis.

Figure S3. GSEA of the GSE63514 dataset.

(A-C) Enrichment of stem cell-related gene sets in tissues of LSIL (A), HSIL (B) and CC (C) compared with those in normal cervix tissues. The blue grid indicates the WONG_EMBRYONIC_STEM_CELL_CORE gene set. (D-F) Enrichment of stem cell differentiation-related gene sets in tissues of LSIL (D), HSIL (E) and CC (F) compared with those in normal cervix tissues. The red grid indicates the BOSCO_EPITHELIAL_DIFFERENTIATION_MODULE gene set. GSEA, Gene Set

Enrichment Analysis; LSIL, low-grade squamous intraepithelial lesions; HSIL, high-grade squamous intraepithelial lesions; CC, cervical cancer.

Figure S4. Characteristics of TIC stemness and differentiation derived from PIWIL2-initiated cellular reprogramming.

SP cells were analyzed using FACS in HaCaT-Lenti, HaCaT-PIWIL2, and HaCaT-PIWIL2-shPDK1 cells (A, B). CD326+ cells can differentiate to CD326- cancer cells (C) and CD31+ vascular endothelial cells (D) in xenograft tumors derived from sorted CD326+ cells, which was measured using FACS and immunofluorescence, respectively. TIC, tumor-initiating cell; SP, side population; PDK1, pyruvate dehydrogenase kinase 1; FACS, fluorescence-activated cell sorting.

Figure S5. The tumor suppression of PDK1 on tumorigenicity *in vivo*.

(A-C) Six weeks after cells were transplanted subcutaneously in mice, HaCaT-PIWIL2 cells formed evident tumors whereas HaCaT cells exhibited no tumorigenicity. HaCaT-PIWIL2 cell tumorigenicity was completely suppressed by PDK1 knockdown. (D) The xenografts derived from HaCaT-PIWIL2 cell transplantation exhibited higher protein levels of PDK1, C-MYC, SOX2, and phosphorylated STAT3 as those in HaCaT cells. PDK1, pyruvate dehydrogenase kinase 1.

Figure S6. Metabolite analysis via GC–MS in HeLa cells stably transduced with the empty, shPIWIL2, or shPDK1 lentiviral vector.

(A) Base peak chromatogram of HeLa cells stably infected with the empty, shPIWIL2 or shPDK1 lentiviral vector. (B) PLS-DA was used to distinguish the differences in the metabolites. (C-D) Boxplot showing the differences in the abundances of products of different cellular energy metabolism pathways between HeLa and HeLa-shPIWIL2 cells (C) and between HeLa and HeLa-shPDK1 cells (D). The data are presented as the means ± SDs. * $P < 0.05$, ** $P < 0.01$. GC–MS, gas chromatography-mass spectrometry; PDK1, pyruvate dehydrogenase kinase 1; PLS-DA, Partial Least Squares

Discrimination Analysis.

Figure S7. Apoptosis in cervical cancer cells evaluated by FACS.

HeLa and SiHa cells were treated with DCA and DDP alone or in combination for 48 h, after which apoptosis was evaluated via FACS using Annexin V-633 and PI staining. FACS, fluorescence-activated cell sorting; DCA, dichloroacetate; DDP, diamminedichloroplatinum.

Figure S8. LIN28A/B regulate aerobic glycolysis and maintain stemness via let-7.

(A) HaCaT cells overexpressing LIN28A or LIN28B exhibited upregulation of LDHA, CK17 and SOX2. (B) HaCaT cells transfected with the let-7g or -7i antagomir exhibited upregulation of LDHA and CK17. (C) LIN28A-overexpressing HaCaT cells transfected with the let-7g or -7i agomir exhibited reduced LDHA expression but upregulated involucrin expression. (D-E) HaCaT cells with stable overexpression of PIWIL2 or with subsequent shRNA-mediated silencing of LIN28A were generated, and glucose uptake (D) and lactate production (E) were then measured in these cells. LDHA, lactate dehydrogenase A.

Figure S9. Human phosphorylation pathway profiling array map

This array contains 18 different phosphorylated human proteins crucial to the AKT pathway, POS (red background) and NEG (blue background), which were used for normalization and determining the level of non-specific binding of the samples, respectively. POS, positive control spot; NEG, negative control spots.

Figure S10. Cellular reprogramming and AKT pathway activation in HCBC with stably overexpressed E6 or PIWIL2.

Apparent upregulation of LIN28A, cellular reprogramming factors (C-MYC, SOX2, OCT4, and NANOG) and stem cell marker CK17 was exhibited in HCBC after stable transfection with PIWIL2 (A) or E6 (B). Overexpression of PIWIL2 also activated AKT pathway in HCBC (C). HCBC, human cervical basal epithelial cells.

Figure S11. Evaluation of gene knocking-down efficiency of specific shRNAs. Three specific shRNAs were designed for silencing each target gene of PDK1, PIWIL2 and LIN28A and transfected with lentiviral vectors whereas the negative control was a scrambled shRNA sequence without specific gene degradation effects. The protein levels of PDK1 (A), PIWIL2 (B) and LIN28A (C) were evaluated by western blotting; the one with the highest knocking-down efficiency was selected for further experiments. PDK1, pyruvate dehydrogenase kinase 1.

Figure S1

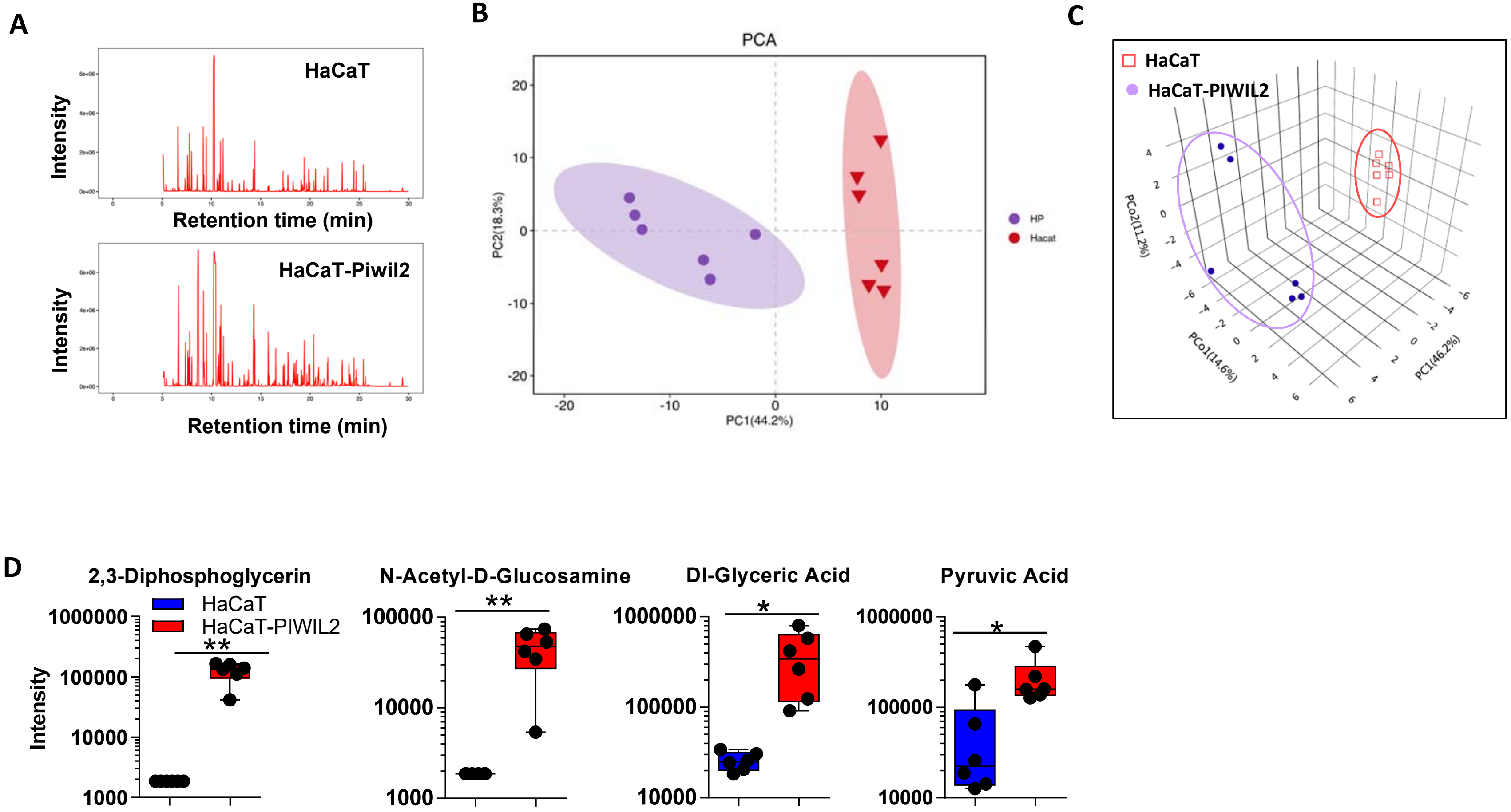

A
HaCaT
Intensity
Retention time (min)
HaCaT-Piwil2
Intensity
Retention time (min)
B
PCA
PC2(18.3%)
PC1(44.2%)
HP
Hacat
C
HaCaT
HaCaT-PIWIL2
PCo2(11.2%)
PCo1(14.6%)
PC1(46.2%)
D
2,3-Diphosphoglycerin
HaCaT
HaCaT-PIWIL2
Intensity
**
N-Acetyl-D-Glucosamine
**
Dl-Glyceric Acid
*
Pyruvic Acid
*

Figure S2

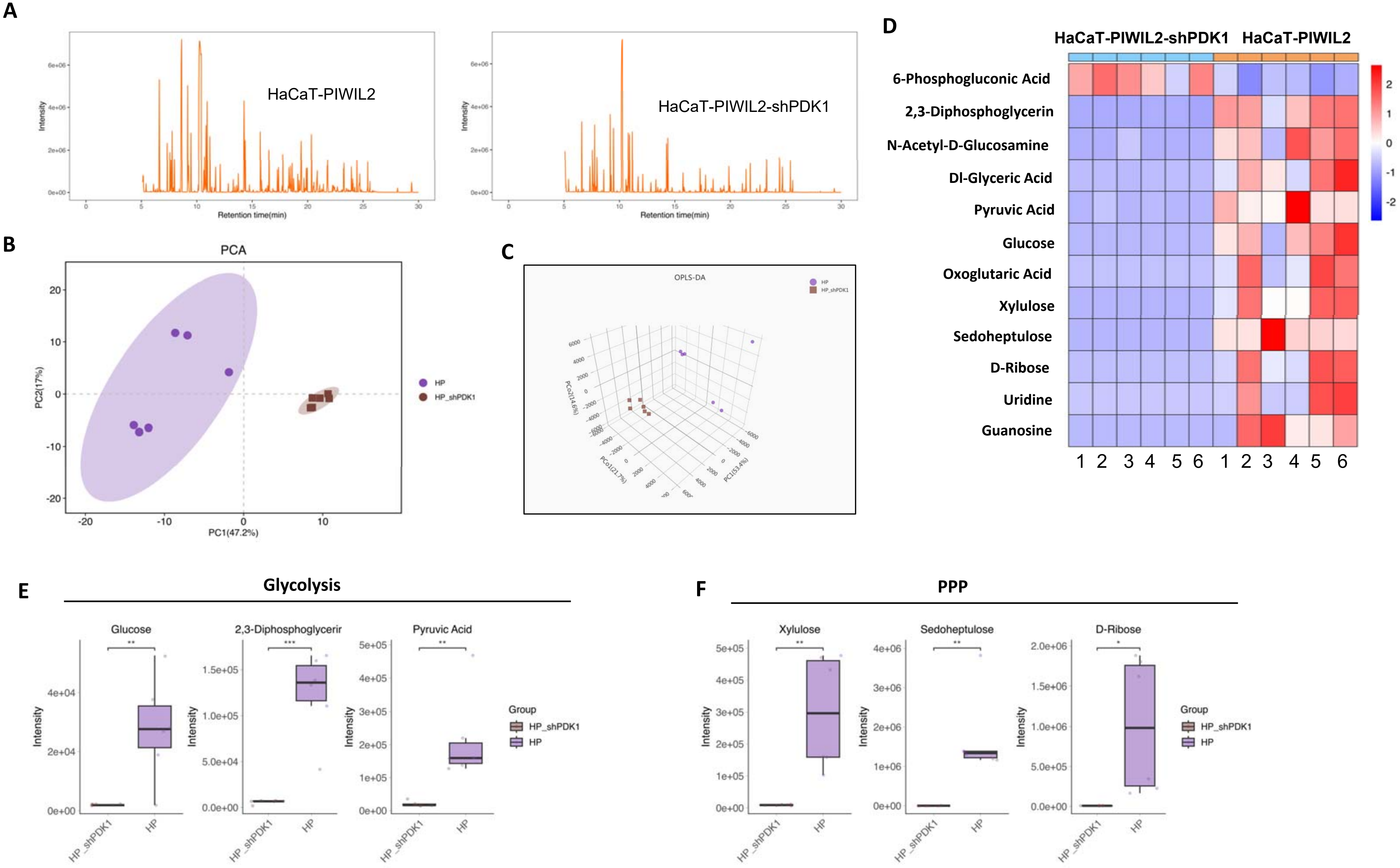

A
HaCaT-PIWIL2
HaCaT-PIWIL2-shPDK1
Retention time(min)
Intensity
B
PCA
PC1(47.2%)
PC2(17%)
HP
HP_shPDK1
C
OPLS-DA
D
HaCaT-PIWIL2-shPDK1
HaCaT-PIWIL2
6-Phosphogluconic Acid
2,3-Diphosphoglycerin
N-Acetyl-D-Glucosamine
Dl-Glyceric Acid
Pyruvic Acid
Glucose
Oxoglutaric Acid
Xylulose
Sedoheptulose
D-Ribose
Uridine
Guanosine
E
Glycolysis
Glucose
2,3-Diphosphoglycerir
Pyruvic Acid
Group
HP_shPDK1
HP
F
PPP
Xylulose
Sedoheptulose
D-Ribose

Figure S3

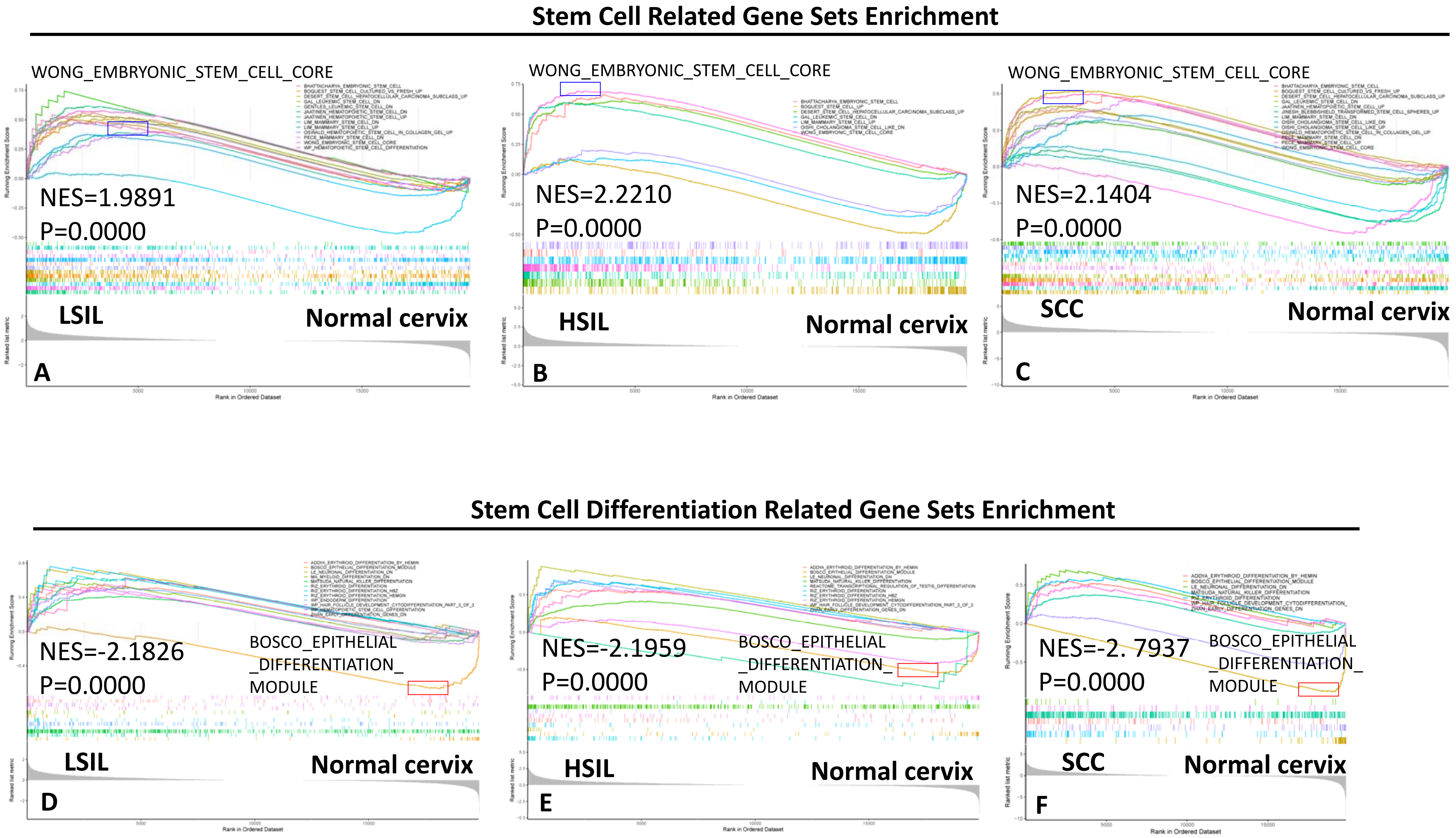
Stem Cell Related Gene Sets Enrichment
WONG_EMBRYONIC_STEM_CELL_CORE
NES=1.9891
P=0.0000
LSIL
Normal cervix
A
WONG_EMBRYONIC_STEM_CELL_CORE
NES=2.2210
P=0.0000
HSIL
Normal cervix
B
WONG_EMBRYONIC_STEM_CELL_CORE
NES=2.1404
P=0.0000
SCC
Normal cervix
C
Stem Cell Differentiation Related Gene Sets Enrichment
NES=-2.1826
P=0.0000
BOSCO_EPITHELIAL
DIFFERENTIATION
MODULE
LSIL
Normal cervix
D
NES=-2.1959
P=0.0000
BOSCO_EPITHELIAL
DIFFERENTIATION
MODULE
HSIL
Normal cervix
E
NES=-2. 7937
P=0.0000
BOSCO_EPITHELIAL
DIFFERENTIATION
MODULE
SCC
Normal cervix
F

Figure S4

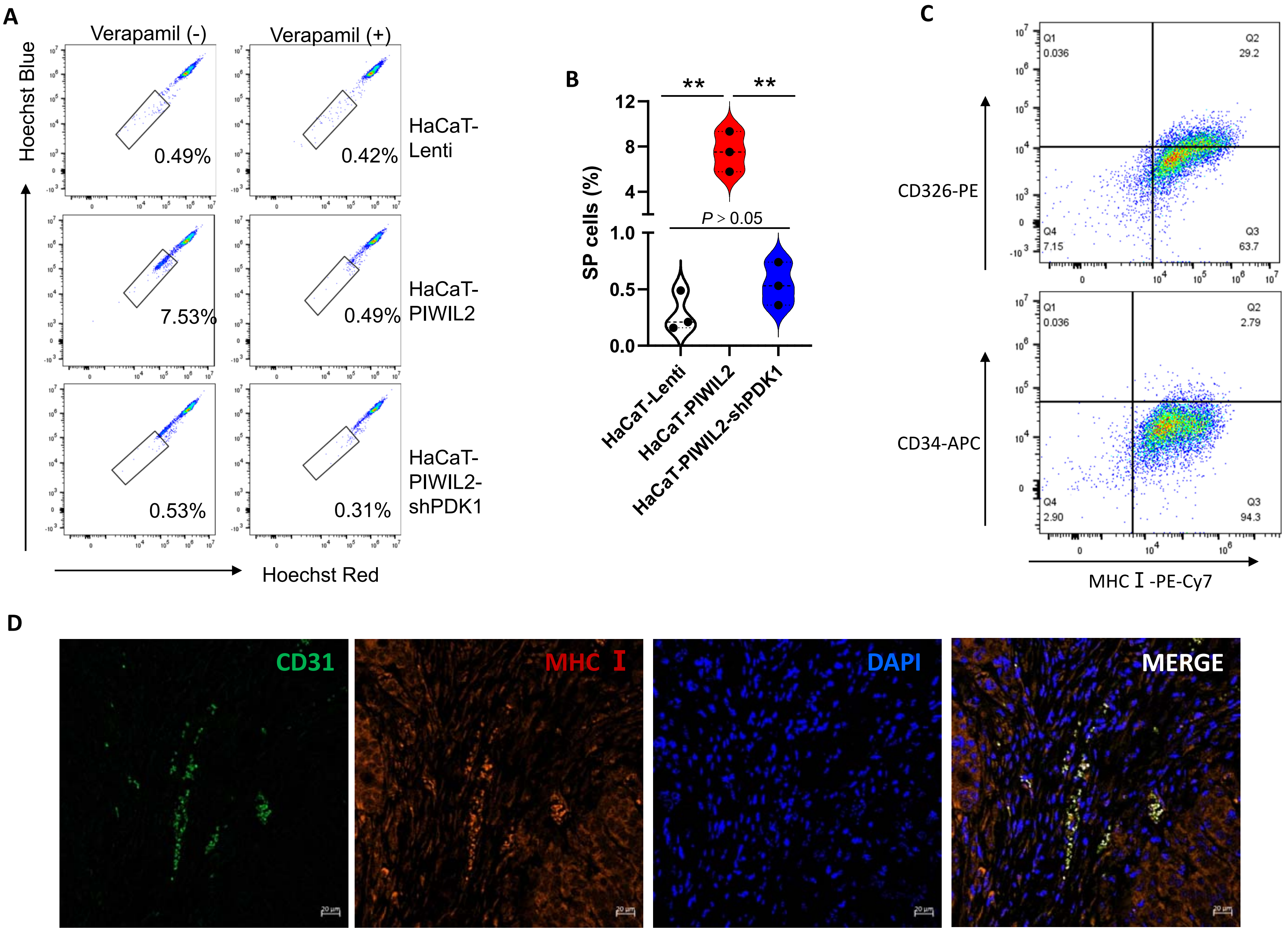
A
Verapamil (-)
Verapamil (+)
Hoechst Blue
Hoechst Red
0.49%
0.42%
HaCaT-Lenti
7.53%
0.49%
HaCaT-PIWIL2
0.53%
0.31%
HaCaT-PIWIL2-shPDK1
B
**
**
P > 0.05
SP cells (%)
HaCaT-Lenti
HaCaT-PIWIL2
HaCaT-PIWIL2-shPDK1
C
CD326-PE
Q1 0.036
Q2 29.2
Q4 7.15
Q3 63.7
CD34-APC
Q1 0.036
Q2 2.79
Q4 2.90
Q3 94.3
MHC Ⅰ -PE-Cy7
D
CD31
MHC Ⅰ
DAPI
MERGE

Figure S5

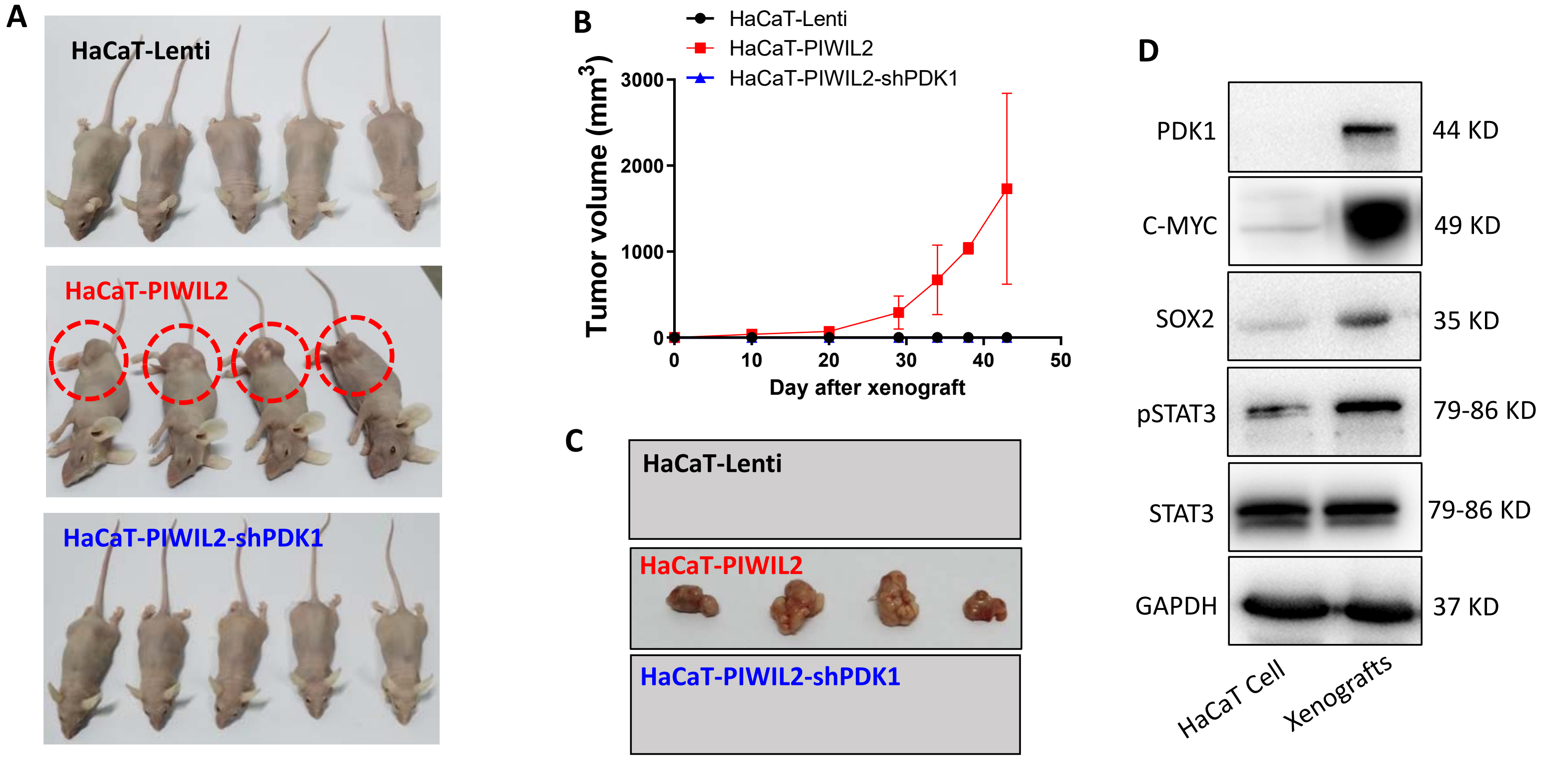

A
HaCaT-Lenti
HaCaT-PIWIL2
HaCaT-PIWIL2-shPDK1
B
HaCaT-Lenti
HaCaT-PIWIL2
HaCaT-PIWIL2-shPDK1
Tumor volume (mm3)
3000
2000
1000
0
0
10
20
30
40
50
Day after xenograft
C
HaCaT-Lenti
HaCaT-PIWIL2
HaCaT-PIWIL2-shPDK1
D
PDK1
44 KD
C-MYC
49 KD
SOX2
35 KD
pSTAT3
79-86 KD
STAT3
79-86 KD
GAPDH
37 KD
HaCaT Cell
Xenografts

Figure S6

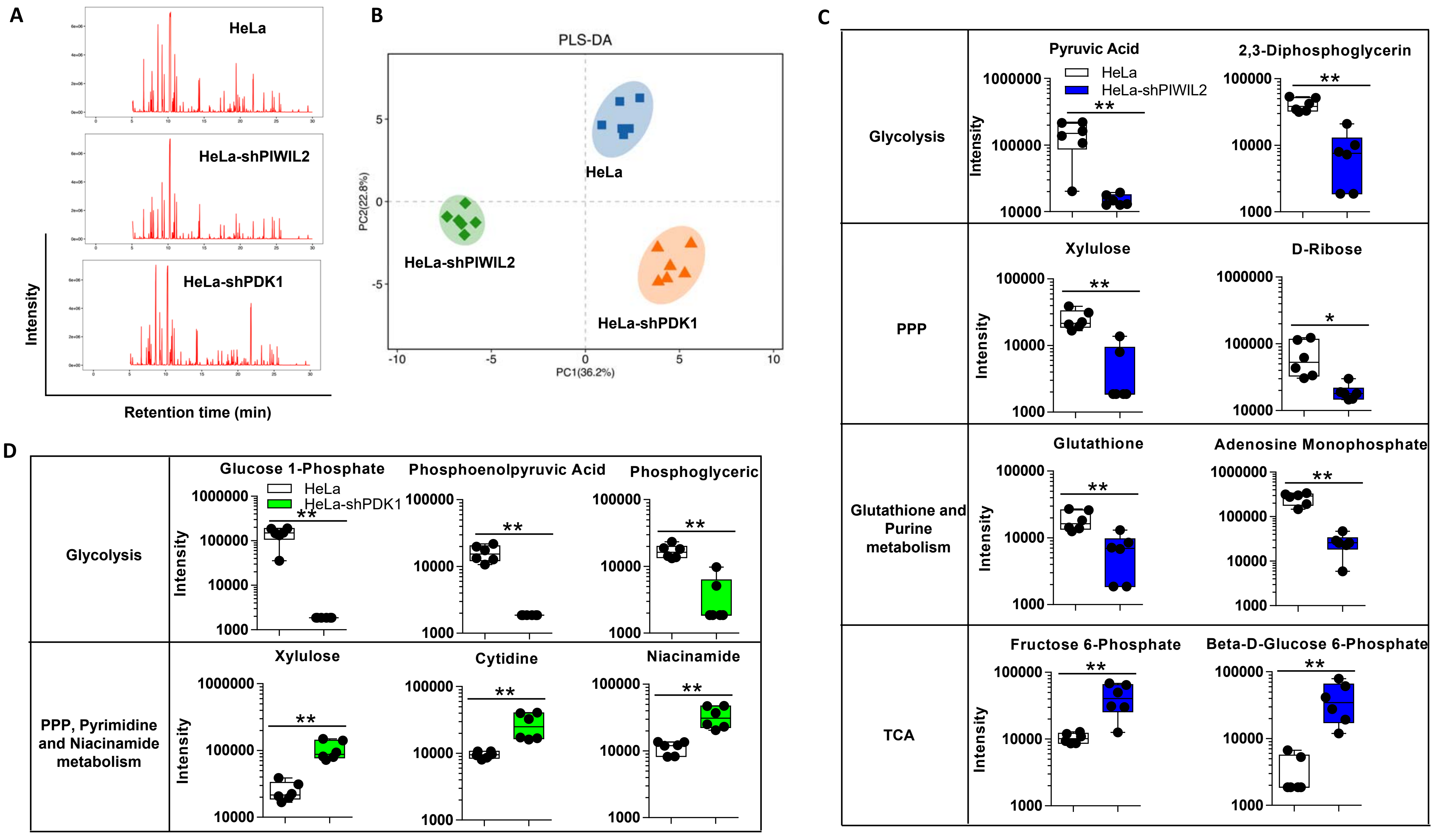

A
HeLa
HeLa-shPIWIL2
HeLa-shPDK1
Intensity
Retention time (min)
B
PLS-DA
HeLa
HeLa-shPIWIL2
HeLa-shPDK1
PC1(36.2%)
PC2(22.8%)
C
Glycolysis
Pyruvic Acid
2,3-Diphosphoglycerin
HeLa
HeLa-shPIWIL2
Intensity
PPP
Xylulose
D-Ribose
Glutathione and Purine metabolism
Glutathione
Adenosine Monophosphate
TCA
Fructose 6-Phosphate
Beta-D-Glucose 6-Phosphate
D
Glycolysis
Glucose 1-Phosphate
Phosphoenolpyruvic Acid
Phosphoglyceric
HeLa
HeLa-shPDK1
PPP, Pyrimidine and Niacinamide metabolism
Xylulose
Cytidine
Niacinamide

Figure S7

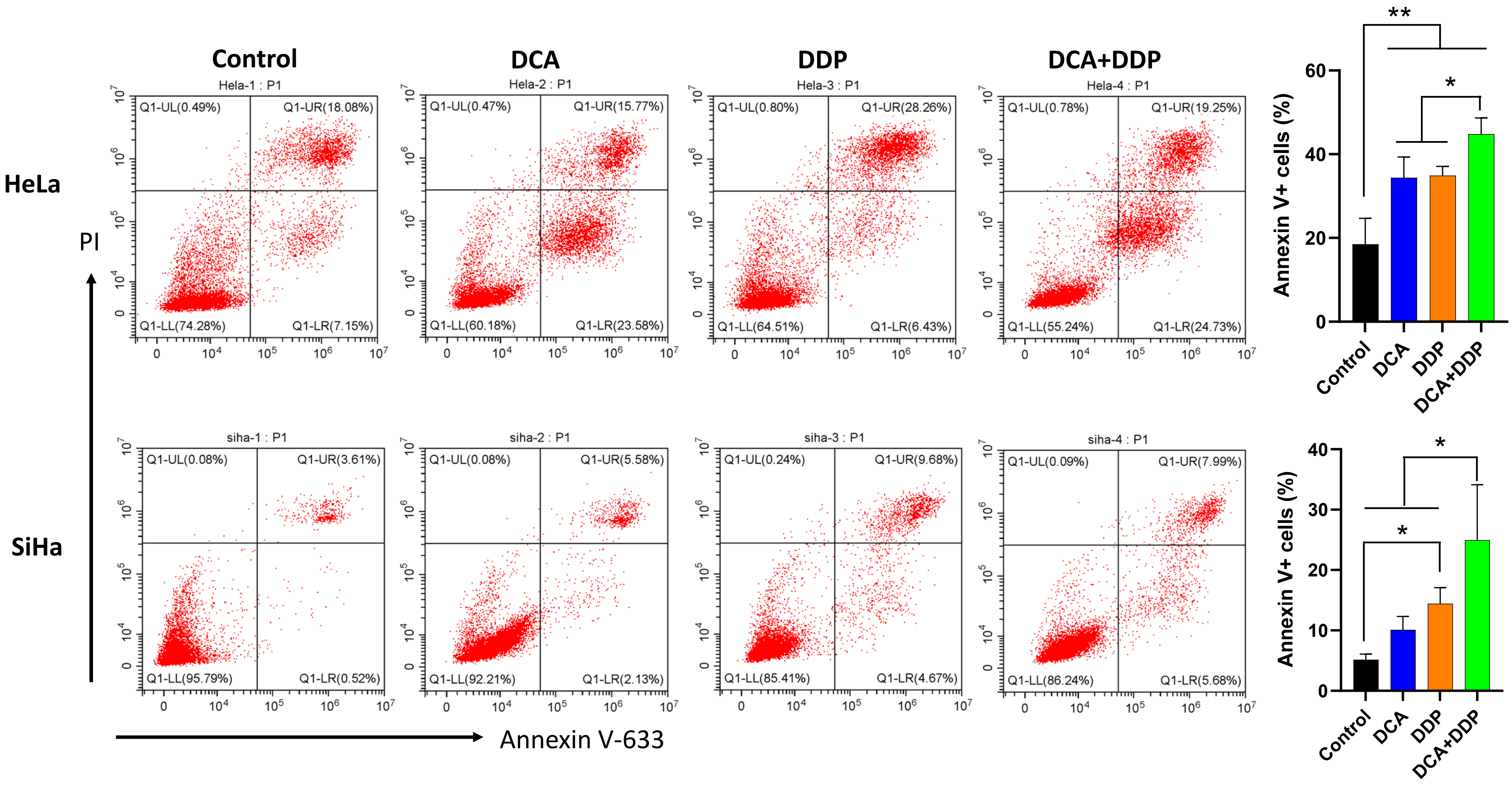
Control
DCA
DDP
DCA+DDP
HeLa
SiHa
PI
Annexin V-633
Hela-1 : P1
Q1-UL(0.49%)
Q1-UR(18.08%)
Q1-LL(74.28%)
Q1-LR(7.15%)
Hela-2 : P1
Q1-UL(0.47%)
Q1-UR(15.77%)
Q1-LL(60.18%)
Q1-LR(23.58%)
Hela-3 : P1
Q1-UL(0.80%)
Q1-UR(28.26%)
Q1-LL(64.51%)
Q1-LR(6.43%)
Hela-4 : P1
Q1-UL(0.78%)
Q1-UR(19.25%)
Q1-LL(55.24%)
Q1-LR(24.73%)
siha-1 : P1
Q1-UL(0.08%)
Q1-UR(3.61%)
Q1-LL(95.79%)
Q1-LR(0.52%)
siha-2 : P1
Q1-UL(0.08%)
Q1-UR(5.58%)
Q1-LL(92.21%)
Q1-LR(2.13%)
siha-3 : P1
Q1-UL(0.24%)
Q1-UR(9.68%)
Q1-LL(85.41%)
Q1-LR(4.67%)
siha-4 : P1
Q1-UL(0.09%)
Q1-UR(7.99%)
Q1-LL(86.24%)
Q1-LR(5.68%)
Annexin V+ cells (%)
Control
DCA
DDP
DCA+DDP
**
*

Figure S8

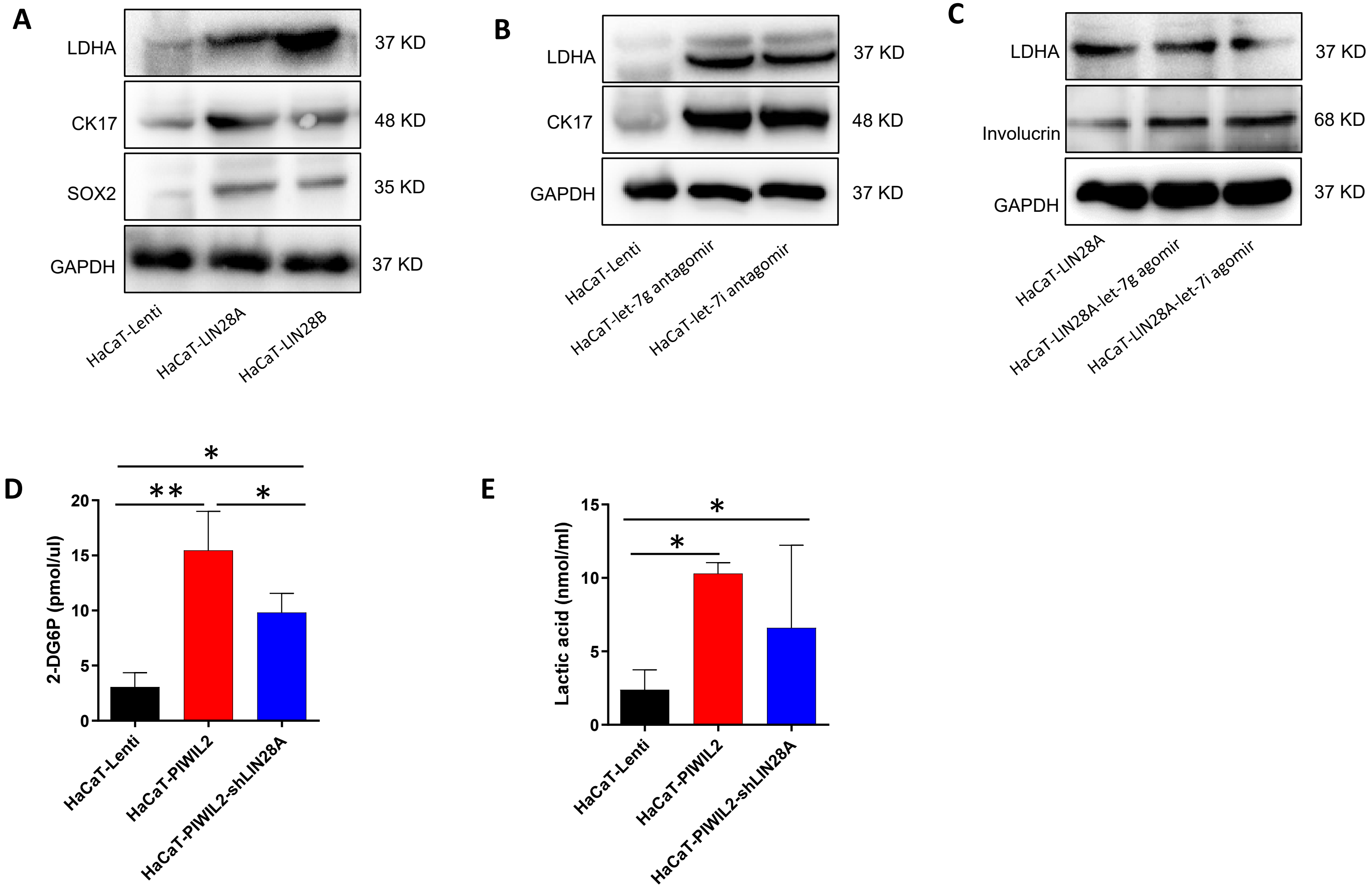
A
LDHA
CK17
SOX2
GAPDH
37 KD
48 KD
35 KD
37 KD
HaCaT-Lenti
HaCaT-LIN28A
HaCaT-LIN28B
B
LDHA
CK17
GAPDH
37 KD
48 KD
37 KD
HaCaT-Lenti
HaCaT-let-7g antagomir
HaCaT-let-7i antagomir
C
LDHA
Involucrin
GAPDH
37 KD
68 KD
37 KD
HaCaT-LIN28A
HaCaT-LIN28A-let-7g agomir
HaCaT-LIN28A-let-7i agomir
D
2-DG6P (pmol/ul)
0
5
10
15
20
**
*
*
HaCaT-Lenti
HaCaT-PIWIL2
HaCaT-PIWIL2-shLIN28A
E
Lactic acid (nmol/ml)
0
5
10
15
*
*
HaCaT-Lenti
HaCaT-PIWIL2
HaCaT-PIWIL2-shLIN28A

Figure S9

Human phosphorylation pathway profiling array map

1<br>2	Positve Control Spot	Positve Control Spot	Negative Control Spot	Negative Control Spot	Akt (p-Ser473)	AMPK (p-Thr172)	BAD (p-Ser112)	4E-BP1 (p-Thr36)
3<br>4	ERK1 (p-T202/Y204) ERK2 (p-Y185/Y187)	GSK3a (p-Ser21)	GSK3b (p-Ser9)	mTOR (p-Ser2448)	P27 (p-Thr198)	P53 (p-Ser15)	S6K (p-Thr421/Ser424)	PDK1 (p-Ser241)
5<br>6	PRAS40 (p-Thr246)	PTEN (p-Ser380)	Raf-1 (p-Ser301)	S6 (p-Ser235/236)	RSK1 (p-Ser380)	RSK2 (p-Ser386)	Negative Control Spot	Positve Control Spot

Figure S10

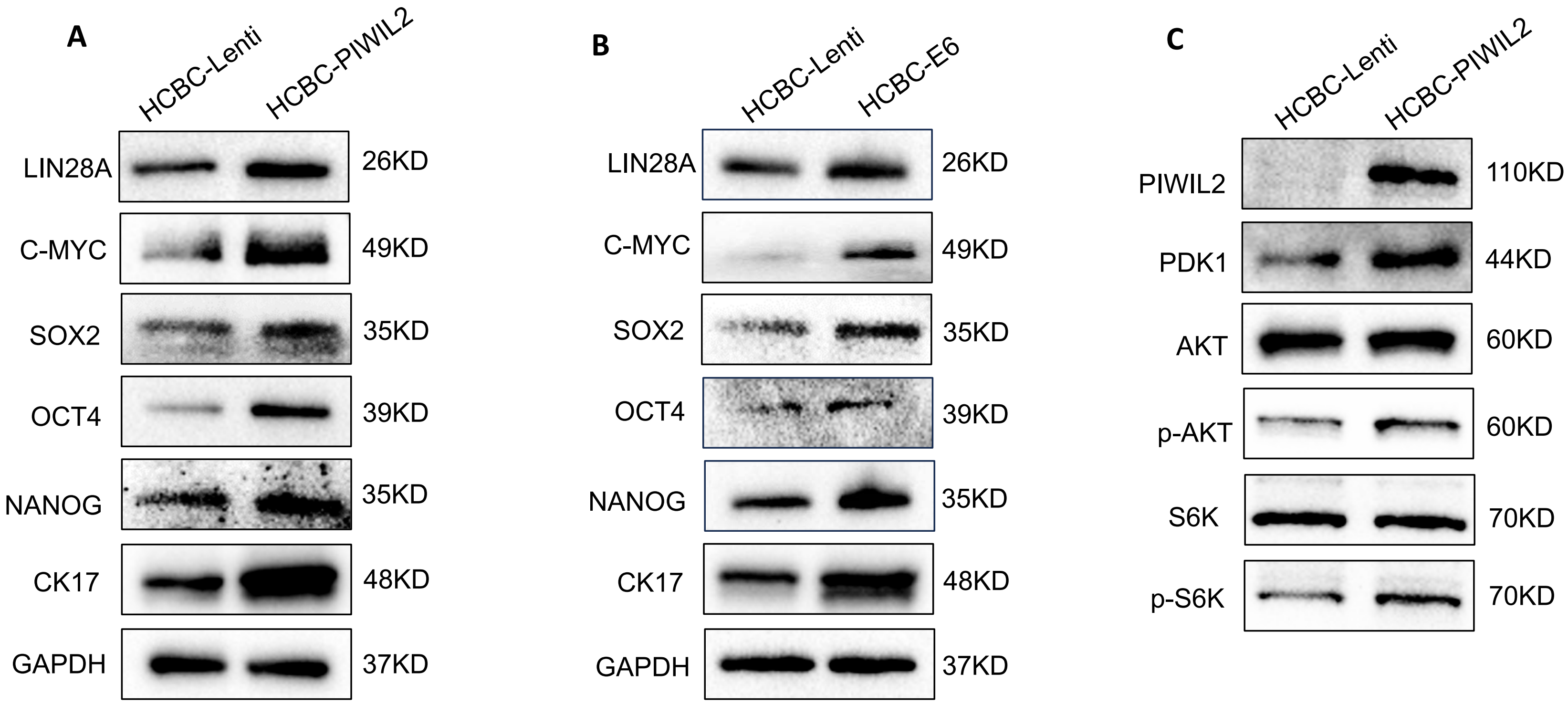
A
HCBC-Lenti
HCBC-PIWIL2
LIN28A
26KD
C-MYC
49KD
SOX2
35KD
OCT4
39KD
NANOG
35KD
CK17
48KD
GAPDH
37KD
B
HCBC-Lenti
HCBC-E6
LIN28A
26KD
C-MYC
49KD
SOX2
35KD
OCT4
39KD
NANOG
35KD
CK17
48KD
GAPDH
37KD
C
HCBC-Lenti
HCBC-PIWIL2
PIWIL2
110KD
PDK1
44KD
AKT
60KD
p-AKT
60KD
S6K
70KD
p-S6K
70KD

Figure S11

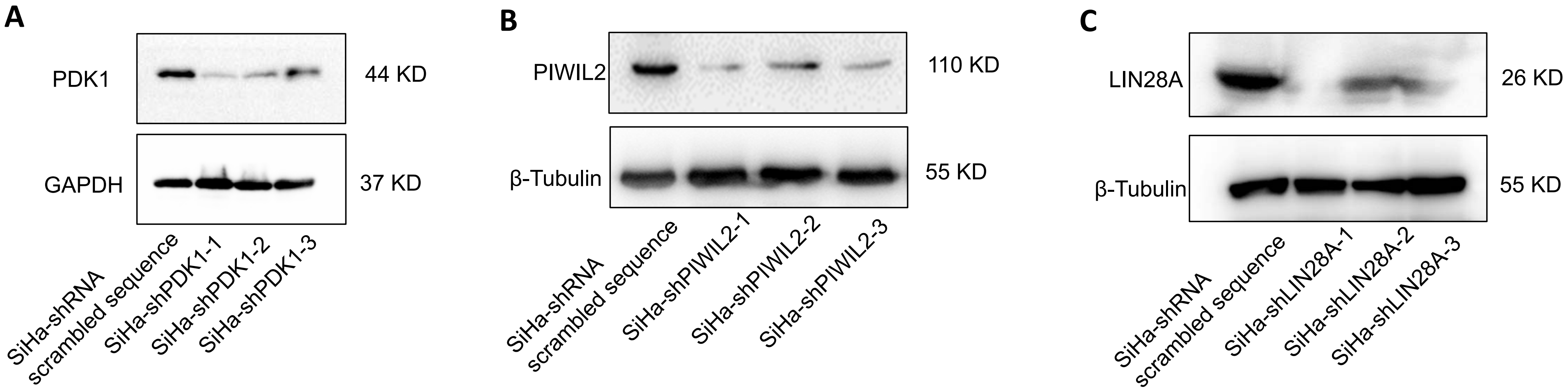
A
PDK1
44 KD
GAPDH
37 KD
SiHa-shRNA scrambled sequence
SiHa-shPDK1-1
SiHa-shPDK1-2
SiHa-shPDK1-3
B
PIWIL2
110 KD
β-Tubulin
55 KD
SiHa-shRNA scrambled sequence
SiHa-shPIWIL2-1
SiHa-shPIWIL2-2
SiHa-shPIWIL2-3
C
LIN28A
26 KD
β-Tubulin
55 KD
SiHa-shRNA scrambled sequence
SiHa-shLIN28A-1
SiHa-shLIN28A-2
SiHa-shLIN28A-3
